# Unveiling the interactions between design optimization, modeling strategies, and spatial analysis in genomic selection

**DOI:** 10.1007/s00122-026-05285-8

**Published:** 2026-08-28

**Authors:** Javier Fernández-González, Jordi Comadran Trabal, Bertrand Haquin, Alix Allard, Eliette Combes, Karine Bernard, Julio Isidro y Sánchez

**Affiliations:** 1 Centro de Biotecnologia y Genómica de Plantas (CBGP, UPM-INIA), Universidad Politécnica de Madrid (UPM) - Instituto Nacional de Investigación y Tecnologia Agraria y Alimentaria (INIA), Campus de Montegancedo-UPM, 28223 Pozuelo de Alarcón, Madrid Spain; 2 Syngenta, Saint-Sauveur, France

## Abstract

***Key message*:**

Partially-replicated designs are more efficient than fully-replicated ones. They can be further enhanced through optimization and present positive synergy with fully-efficient models and 2D P-splines.

**Abstract:**

In plant breeding, traditional field trials rely on balanced designs with high replication to ensure robust breeding value predictions. However, genomic technologies leveraging genomic relationship matrices have reduced the need for extensive replication, favoring partially-replicated (p-rep) designs. These designs, while effective, pose statistical challenges due to low replication and imbalance. To address these, we conducted simulations using datasets from maize, sunflower, and oat, evaluating interactions between experimental designs and modeling strategies across varying field sizes. Classical and p-rep designs, generated via randomization or optimization, were compared, using a novel, faster implementation of the CDmean optimization criterion developed to overcome computational limits. Modeling strategies included single-stage models, unweighted and fully efficient two-stage analysis, and spatial analysis with blocking structures or high-resolution 2D P-splines. Our findings showed p-rep designs consistently outperformed classical designs in predicting unobserved lines, achieving 1–25% higher accuracies depending on the scenario. Within observed training sets, p-rep designs exhibited slightly lower accuracy than classical designs, but it is compensated with increased selection intensity. Design optimization enhanced accuracy by  1% over randomized p-rep designs, while 2D P-splines improved accuracy by 1–2% when paired with single-stage or fully efficient models.

**Supplementary Information:**

The online version contains supplementary material available at https://doi.org/10.1007/s00122-026-05285-8.

## Introduction

In genomic selection, the allele serves as the fundamental unit of selection, allowing plant breeders to replicate genetic information across related individuals (Lorenz et al. 2011). This shift has revolutionized plant breeding by enabling more efficient experimental designs and improving selection accuracy (Alemu et al. 2024). Field trials are indispensable for genomic selection, serving both as a means to directly evaluate germplasm and as training sets (TRS) to calibrate genomic prediction (GP) models, which predict the performance of untested genotypes in a test set (TS) (Meuwissen et al. 2001). Traditional experimental designs, such as randomized complete block designs (RCBD), rely on replication and blocking to manage spatial variation and ensure reliable estimates of genetic effects (Fisher 1926; Casler 2015). While effective for small trials, fully replicated designs limit the number of genotypes tested, reducing selection intensity and the size of TRS datasets, both of which are critical for maximizing genetic gain (Clark et al. 2012; Rincent et al. 2012; Fernández-González et al. 2023).

Partially replicated designs (p-rep) (Cullis et al. 2006) have emerged as a powerful alternative to classical designs. By reducing replication, these designs allow for more genotypes to be tested in the same field size, increasing selection intensity while maintaining sufficient degrees of freedom for genetic effect estimation. Genomic information complements these designs through the genomic relationship matrix, which captures genetic covariance among individuals (Endelman et al. 2014). This enables low-replication designs to achieve similar predictive power to fully replicated designs by leveraging shared genetic information across related individuals. Consequently, p-rep and augmented designs (Federer 1956) are increasingly popular in modern plant breeding programs. It is important to note that augmented designs differ from p-rep in the fact that the replicated genotypes are standard varieties instead of a sample from the experimental entries (Zystro et al. 2018). If these standard varieties are strongly related to the rest of the population, augmented and p-rep designs are functionally identical for genomic prediction purposes. Otherwise, we may obtain slightly lower accuracy from augmented designs, as the weak relationships could limit the propagation of information from replicated entries to unreplicated ones. More research is needed to verify this.

Beyond replication, the availability of genomic data facilitates the optimization of genotype placement in the field. By strategically separating related genotypes, optimized designs minimize confounding spatial effects and improve the estimation of genetic effects (Feoktistov et al. 2017). Optimization is commonly guided by prediction error variance (PEV)-based criteria, such as A-optimality (Cullis et al. 2006) and CDmean, which improve accuracy while only incurring increased computational time. However, despite their promise, the interaction between optimized experimental designs and downstream statistical models remains understudied. This is critical, as the benefits of optimization likely depend on the choice of spatial analysis and the integration of genomic information during the analysis.

To analyze field data, plant breeders frequently rely on linear mixed models. Single-stage (SS) models are the statistical gold standard, simultaneously accounting for spatial variation, environmental effects, and genomic relationships in a single framework (Welham et al. 2010). While powerful, SS models are computationally demanding and lack flexibility in handling location-specific spatial structures (Piepho et al. 2012). Two-stage models address these limitations, with unweighted two-stage (UNW) models separating spatial and genomic analysis into distinct stages for improved computational efficiency. However, UNW models often fail to fully capture design optimization benefits due to the exclusion of genomic information in the first stage. Fully-efficient two-stage (FE) models overcome this limitation by incorporating estimation error variance (EEV) from the first stage, achieving results comparable to SS models while retaining computational flexibility (Piepho et al. 2012; Endelman 2023; Fernández-González and Isidro y Sánchez, J. 2025).

High-resolution spatial analysis, such as two-dimensional penalized splines (2D p-splines), is also critical for optimized designs, as they model fine-scale spatial trends that cannot be captured by traditional blocking structures (Eilers et al. 2015; Cappa et al. 2019). These methods can be particularly effective when paired with optimized designs, where genotype placement is finely tuned at the plot level. Thus, both the choice of experimental design and the analytical approach are key factors influencing the accuracy of genetic effect estimation and genomic predictions.

This study evaluates the combined impact of experimental design and statistical modeling on GP accuracy. Specifically, we aim to: (i) compare the performance of fully-replicated and partially-replicated designs, including randomized and optimized layouts, (ii) assess the effectiveness of spatial modeling approaches, such as blocking structures and 2D p-splines, in conjunction with different design strategies, and (iii) investigate how statistical models, including SS, FE, and UNW, interact with experimental designs and spatial analysis to influence the accuracy of genetic effect estimation within the TRS and prediction accuracy for the TS.

## Materials and methods

The experimental methodology used in this work, outlined in Fig. 1, starts with empirical marker data from three different datasets (maize, sunflower, and oat). From these populations, a random split is performed to divide them into a TRS and a TS. Then, the phenotype of the TRS is simulated in different scenarios, focusing on crop yield. Yield was chosen for its known low heritability, making the spatial effects more prominent and maximizing the differences between high-replication and low-replication designs (Zystro et al. 2018). A wide range of field sizes was tested, with each field having 2 or 3 blocks, and each block having 25, 50, 100, 150, or 200 plots. That way, the smallest fields had 2×25=50 plots, and the largest ones had 3×200=600 plots. For each field size, four experimental designs were tested, combining fully-replicated or partially-replicated designs with randomized or optimized distribution of the genotypes in the field. The simulated phenotypes of the TRS were used to train six different models in one or two stages, utilizing several spatial analyses. Finally, the model predictions were correlated with the simulated breeding and genotypic values of the TRS and TS to compute accuracy, allowing for the evaluation of which experimental design and modeling strategy is optimal. The construction of the experimental designs was replicated 30 times, and for each one, 10 simulations were conducted. Therefore, the simulations and models were replicated a total of 30×10=300 times.

**Fig. 2:**
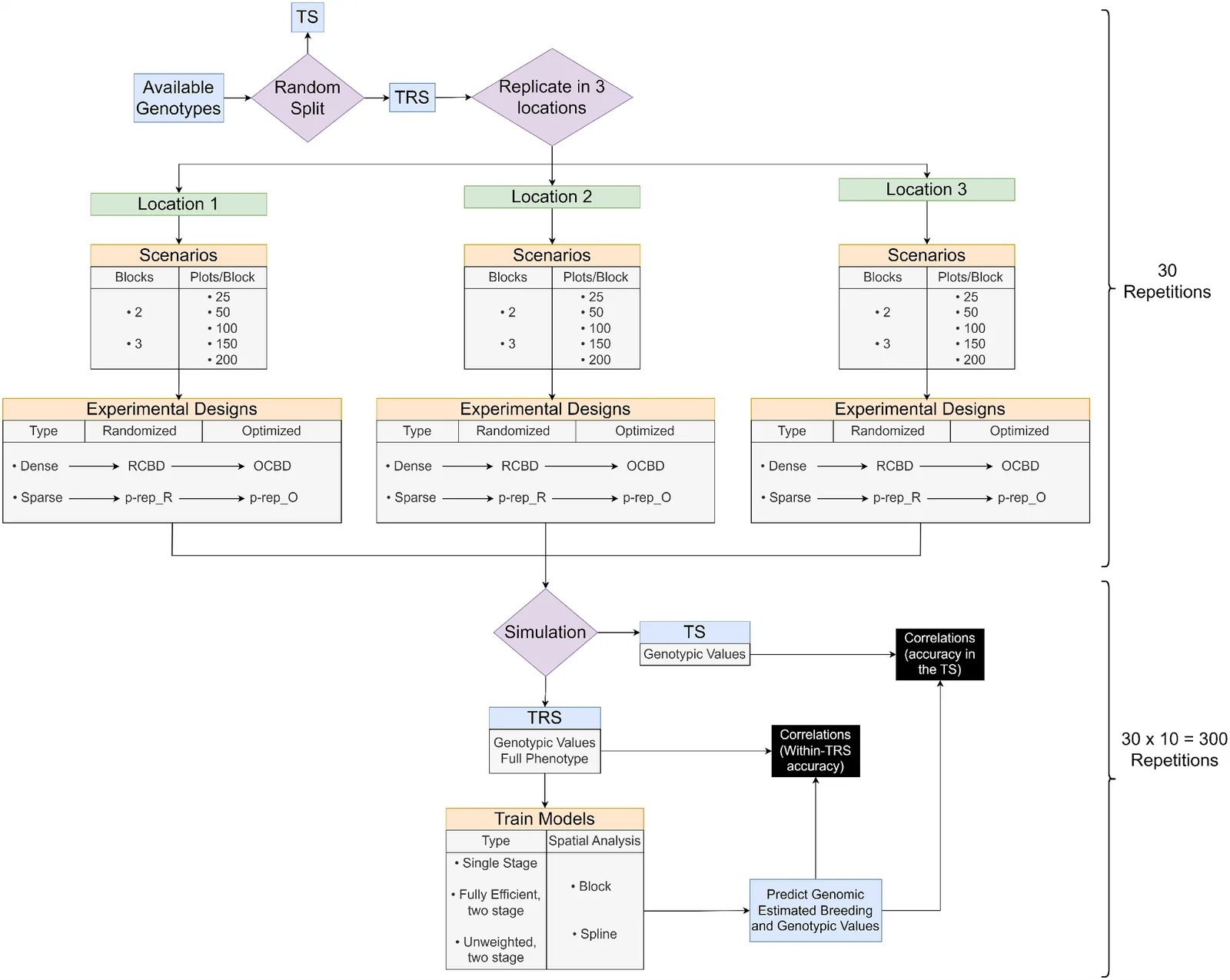
Summary of the experimental procedure followed in this work. A training set (TRS) is tested in field sizes with different experimental designs: randomized complete block design (RCBD); optimized complete block design (OCBD); partially replicated, randomized designs (p-rep_R); partially replicated, optimized designs (p-rep_O). The simulated phenotypes are used to train six different models and their predictions are evaluated through the correlation with the simulated breeding and genotypic values of the TRS and test set (TS)

### Materials

We based the simulations on three empirical datasets: maize hybrids from a full-diallel, sunflower hybrids from a testcross design, and inbred oat lines from a highly admixed population with three main subpopulations. Marker data from these datasets served as the foundation for the simulation. When available, phenotypic data were partitioned into variance components and used as simulation parameters; otherwise, we sourced this information from the literature, as detailed in Appendix 2. The resulting heritability values for the datasets are displayed in Table 1.

**Table 1:** Target heritability used in the simulations for the three datasets. Narrow sense heritability ($h^2$) and broad sense heritability ($H^2$) are displayed

Dataset	$h^2$	$H^2$
Maize	0.204	0.355
Sunflower	0.153	0.203
Oat	0.444	0.640

#### Maize dataset

We utilized a population of 391 maize inbred lines that were genotyped with 244,781 single nucleotide polymorphisms (SNPs) (Hirsch et al. 2014). A subset of 40 diverse and representative parental lines was selected using the Avg_GRM_self algorithm (Fernández-González et al. 2023). A full diallel was conducted, and the genotypes of the resulting 780 hybrids were predicted as the average of the fully inbred parental genotypes. These hybrid genotypes formed the population for the simulations.

Since no phenotypic records were available for the hybrids, we extracted variance components describing yield variability in maize hybrids from the literature (Hallauer et al. 2010; Coutiño-Estrada and Vidal-Martínez 2006; Carvalho et al. 2017; Ferreira Coelho et al. 2021; Yue et al. 2022) to use as simulation parameters. Further details can be found in Appendix 2.

#### Sunflower dataset

Syngenta provided a dataset of testcross sunflower hybrids, comprising 224 hybrids genotyped with 14755 SNPs markers after quality control with the snpReady R package (Granato and Fritsche-Neto 2018). The hybrids were tested across 47 environments over three years, using a RCBD with two blocks per year. Not all hybrids were tested each year. We fitted a model to extract variance components and validated them by comparing them with the literature (Hu et al. 2010; Ahmed et al. 2020; Khan 2001). Additional details are available in Appendix 2.

#### Oat dataset

We used a public oat dataset (Canales et al. 2021), which includes 699 inbred lines from three subpopulations with varying degrees of admixture. The lines were genotyped using 17,288 SNP markers and phenotyped in a RCBD across three environments over two years, with three blocks per environment. Variance components were extracted by fitting a model and incorporating information from the literature (Pfahler 1971; Tanvi and Jindal 2017; Jaiswal et al. 2022), as described in Appendix 2.

### Simulation

All simulations followed the basic equation:

1 $$\begin{aligned} y_{ijk}= &  a_i + g_i + e_j + ae_{ij} + ge_{ij} + p_{jk} + ap_{ijk} \nonumber \\ &  + gp_{ijk} + \epsilon _{ijk} \end{aligned}$$

where $y_{ijk}$ is the phenotype of genotype *i* in plot *k* within the environment *j*. The term $a_i$ represents the additive genetic value, and $g_i$ denotes the non-additive genetic value, which is interpreted as dominance in maize and sunflower hybrids or as epistasis in inbred oat lines. The main environmental effect is $e_j$. The terms $ae_{ij}$ and $ge_{ij}$ capture additive and non-additive genetic interactions with the environment, respectively. The plot effect, $p_{jk}$, represents spatial correlation within the field, while $ap_{ijk}$ and $gp_{ijk}$ describe additive and non-additive genetic interactions with the plot spatial effect. The interaction between the spatial variation of the field and the genotypes is usually not modeled. However, it is known that genotype by environment interaction exists and that the spatial variability of the field has the same nature as the environmental variation across locations, i.e., the genotypes in different parts of the field are subject to slightly different humidity, soil, temperature, wind, etc. Therefore, we argue that a realistic simulation should consider how this variability interacts with the genotypes. Finally, $\epsilon _{ijk}$ represents random residuals. Unlike $p_{jk}$, $ap_{ijk}$, and $gp_{ijk}$, $\epsilon _{ijk}$ contains other sources of variation, such as measurement errors that do not present any spatial correlation.

The simulation scheme is outlined in Fernández-González et al. (2025). In each simulation iteration, 3000 SNPs were sampled from the empirical marker sets and were treated as QTL. Their effects were simulated following the approach of Technow et al. (2012) and Melchinger and Frisch (2023). To model environmental and spatial effects, we first randomly sampled values for environmental covariates. Then, we sampled values for the interaction strength between the genetic effects of each genotype and the environmental covariates and used them to obtain the main environmental effects and the genotype by environment interaction. The generalized effective sample size, as described in Fernández-González et al. (2025), was used to adjust variance components. Target variances from empirical data and literature were treated as expected values rather than fixed constants. In each simulation iteration, a variance value was sampled from a gamma distribution centered on the target variance, with dispersion determined by the effective sample size. This allows for the exploration of likely variance component combinations. More details can be found in Fernández-González et al. (2025) and Appendix 3.

It is important to note that sampling random marker effects for a fixed set of marker scores introduces some limitations into our simulation. Firstly, we are not differentiating between QTL and markers; secondly, we are not simulating the negative correlations between QTL induced by the Bulmer effect (Bulmer 1971). Because of this, if we simulated crosses between the simulated genotypes, their offspring would not be realistic because (i) we would not capture the reduction in accuracy due to recombination between the QTL and the markers, and (ii) genetic variance would rapidly drop under selection due to the sudden introduction of the Bulmer effect. Both of these problems do not apply to our current simulation because we are only simulating phenotypes in field trials within a single generation; we are not simulating crosses between genotypes. A deeper discussion on this topic is available in Appendix 3.

### Experimental designs

For each field size described in Fig. 1, we developed four experimental designs to explore all combinations of partially-replicated and fully-replicated designs with either randomized or optimized genotype allocation in the field. In all designs, genotypes were fully replicated across locations. The difference between partially-replicated and fully-replicated designs lies in whether genotypes are replicated across blocks within each location.

- Randomized Complete Block Design (RCBD): All genotypes are fully replicated across blocks within each location, with randomized positions.
- Optimized Complete Block Design (OCBD): Similar to RCBD, but with optimized rather than randomized genotype distribution within the field. The CDmean optimization method was used.
- Partially Replicated, Randomized Designs (p-rep_R): Some genotypes (replicated entries) are randomly selected and replicated across blocks, while others are tested once per location. Replicated entries constitute 20% of the block size, the minimum needed for consistent spatial analysis based on preliminary testing.
- Partially Replicated, Optimized Designs (p-rep_O): Similar to p-rep_R, but the replicated entries are selected through optimization (Avg_GRM_self criterion), and the positions of all genotypes in the field are optimized rather than randomized using the CD mean.

For the optimized designs, we used two criteria:

#### Minimization of the average genomic relationship (Avg_GRM_self)

This method (Fernández-González et al. 2023) selects a subset of genotypes with minimal genetic relatedness: $Avg\_GRM\_self = \text {mean}(G[\text {Sel},\text {Sel}])$, where *G* is the additive relationship matrix, and $G[\text {Sel},\text {Sel}]$ represents the subset of *G* corresponding to selected individuals ($\text {Sel}$). By minimizing this value, we ensure high diversity and a representative sample of the broader population. We applied this method to select optimal replicated entries in the p-rep_O and to choose 40 representative parental lines from the maize population. The TrainSel heuristic (Akdemir et al. 2021) was used to identify the subset of genotypes that minimized $Avg\_GRM\_self$.

#### Maximization of the mean coefficient of determination (CDmean)

This parametric optimization criterion, typically used for TRS optimization (Laloë 1993; Rincent et al. 2012), is based on genomic data. Here, we present a variation that remains numerically equivalent but allows for the inclusion of an assumed autoregressive spatial structure in the field without increased computational costs. This approach is crucial for optimizing genotype distribution in the field. To achieve this, we directly computed the PEV matrix from the Henderson equations (Henderson 1975) and then derived the coefficient of determination (CD) from it (Laloë 1993; Rincent et al. 2012):

2 $$\begin{aligned} \begin{array}{cc} {\boldsymbol{y}} = X\mu + Z{\boldsymbol{a}} + \boldsymbol{\epsilon } \\ {\boldsymbol{a}} \sim N({\boldsymbol{0}}, G\sigma ^2_a) \\ \boldsymbol{\epsilon } \sim N({\boldsymbol{0}}, R\sigma ^2_\epsilon ) \\ \begin{pmatrix} X'(R\sigma ^2_\epsilon )^{-1}X & X'(R\sigma ^2_\epsilon )^{-1}Z \\ Z'(R\sigma ^2_\epsilon )^{-1}X & Z'(R\sigma ^2_\epsilon )^{-1}Z + (G\sigma ^2_a)^{-1} \end{pmatrix}^{-1}\\ {=} \begin{pmatrix} C_{11} & C_{12} \\ C_{21} & C_{22} {=} PEV \end{pmatrix} \\ CD = (G\sigma ^2_a - PEV) \oslash (G\sigma ^2_a) \\ CDmean = \boldsymbol{w'} diag(CD) \\ \underset{Z}{\textrm{argmin}} \; CDmean \end{array} \end{aligned}$$

where ⊘ refers to the element-wise division. CDmean is based on a model where the phenotype *y* depends on a fixed intercept μ, a random additive genetic effect ${\boldsymbol{a}}$, and residuals $\boldsymbol{\epsilon }$. The design matrix *X* corresponds to the intercept (a vector of ones), and *Z* is the design matrix representing the distribution of genotypes in the field. The variance components $\sigma ^2_a$ and $\sigma ^2_\epsilon$ are commonly set to one (Rincent et al. 2012), which is a standard choice in optimization problems. This setup minimizes the coefficient matrix size from the Henderson equations, which is key for computational efficiency, while incorporating all necessary information for optimization: the genomic relationship matrix (*G*) and the covariance structure for spatial effects in the field (*R*).

Although *R* is often unknown, it can be assumed to follow a two-dimensional, separable autoregressive structure of order one (AR1×AR1) (Gilmour et al. 1997; Stefanova et al. 2009). This creates a correlation matrix *R* that depends on the autocorrelation in rows ($\rho _{rows}$) and columns ($\rho _{\text {cols}}$). For simplicity, we assumed that $\rho = \rho _{\text {rows}} = \rho _{\text {cols}}$. This parameter controls whether the optimization focuses on local neighbors (low ρ values) or globally on the entire field (high ρ values). Preliminary testing showed that the optimal ρ value results in a correlation between the most distant plots of $10^{-3}$, making it dependent only on field size and easy to tune. More details are available in Appendix 4.

The *G* matrix includes all TRS genotypes and can also include TS individuals who will not be phenotyped but are important for prediction, allowing for targeted optimization (Akdemir and Isidro-Sánchez 2019). The *diag*() function extracts the main diagonal of a matrix, and $\boldsymbol{w'}$ is a vector of weights that controls the relative importance of TRS and TS genotypes in the optimization. Additional details are provided in Appendix 5.

We used the TrainSel heuristic (Akdemir et al. 2021) to find the genotype distribution in the field (i.e., the *Z* matrix) that maximized CD_mean.

### Models

We built genomic prediction models on the simulated data and computed their prediction accuracy, using it to evaluate which experimental design and modeling strategy yielded better results.

We used three different types of linear mixed models: a single-stage model (SS), an unweighted two-stage model (UNW), and a fully-efficient two-stage model (FE). All models were fitted with the Sommer R package (Covarrubias-Pazaran 2016). Each model was a simplification of reality, containing fewer terms than those used in the simulations, to mimic the common scenario in which models fitted to empirical data are simpler than the complex biological processes influencing the phenotype. For instance, we simulated genotype by environment interactions, but we did not include them explicitly in the models, letting them be absorbed by the residuals.

All models allow for the prediction of both additive ($\boldsymbol{{\hat{a}}}$) and non-additive ($\boldsymbol{{\hat{g}}}$) genetic values of the TS. Accuracy was calculated for the breeding values ($\boldsymbol{{\hat{a}}}$) and the genotypic values ($\boldsymbol{{\hat{a}}}$ + $\boldsymbol{{\hat{g}}}$) by correlating the true (simulated) values with the model predictions. Please note that the validation scheme outlined in Fig. 1 is slightly different from a classical cross-validation. This was possible because, when working with simulations, the true genotypic values are known, which facilitates accuracy calculation.

#### Single-stage model

This model had the following equation:

3 $$\begin{aligned} {\boldsymbol{y}} = X_e{\boldsymbol{e}} - ({\boldsymbol{F}}b) + W{\boldsymbol{p}} + Z_a{\boldsymbol{a}} + Z_g {\boldsymbol{g}} + \boldsymbol{\epsilon } \end{aligned}$$

where ${\boldsymbol{y}}$ is a vector of phenotypes. ${\boldsymbol{e}}$ is a vector of fixed environmental effects, and ${\boldsymbol{p}}$ represents spatial effects nested within the environment. We tested two alternatives for ${\boldsymbol{p}}$: a fixed block effect or a random effect using 2D p-splines. The latter estimates spatial effects with single plot resolution, with spline penalization allowing for more coefficients than available data points (Rodríguez-Álvarez et al. 2017; Lee et al. 2013; Eilers et al. 2015). Both spatial analyses differ from the autoregressive process used in the simulation to avoid unfair advantages associated with assuming the exact same spatial process (Cappa et al. 2019). We compared block effects with two-dimensional P-splines because they represent markedly different spatial resolutions, providing a clear contrast to assess the impact of spatial modeling. In practical applications, intermediate approaches, such as finer blocking or the inclusion of row and column effects, may also be appropriate.

The vector ${\boldsymbol{a}} \sim N({\boldsymbol{0}}, G\sigma ^2_a)$ represents additive genetic effects, where *G* is the VanRaden (2008) additive genomic relationship matrix. The vector ${\boldsymbol{g}} \sim N({\boldsymbol{0}}, \varSigma \sigma ^2_g)$ represents non-additive effects. For the maize and sunflower hybrid datasets, ${\boldsymbol{g}}$ corresponds to dominance effects, with Σ as the Vitezica et al. (2013) dominance relationship matrix (*D*). For the oat dataset, ${\boldsymbol{g}}$ referred to epistatic effects, with $\varSigma = G \circ G$, where ∘ indicates the Hadamard product (Henderson 1985; Gianola and de Los 2008). Residuals are captured by $\boldsymbol{\epsilon } \sim N({\boldsymbol{0}}, \text {diag}(e) \otimes I)$, with heterogeneous variance across environments. In this work, we define $\text {diag}(e)$ as:

$$\begin{aligned} \text {diag}(e) = \begin{pmatrix} \sigma ^2_{1} & \quad 0 & \quad \dots & \quad 0 \\ 0 & \quad \sigma ^2_{2} & \quad & \quad 0 \\ \vdots & \quad & \quad \ddots & \quad \vdots \\ 0 & \quad 0 & \quad \dots & \quad \sigma ^2_{j} \end{pmatrix}\end{aligned}$$

i.e., a diagonal covariance structure across the *j* environments. The term ${\boldsymbol{F}}b$ (only present for hybrid crops) represents directional dominance, and its inclusion allows dominance values to be non-zero on average; i.e., it allows for the capture of heterosis (b>0) and inbreeding depression b<0. *b* is a fixed regression coefficient, and ${\boldsymbol{F}}$ is a vector of inbreeding coefficients calculated as ${\boldsymbol{F}} = \text {diag}(G)-{\boldsymbol{1}}$. This term is preceded by a minus sign, following the convention from Endelman (2023). The term *Fb* is enclosed in brackets to indicate that it was removed from the oat dataset, as it does not demonstrate dominance. $X_e$, *W*, $Z_a$, and $Z_g$ are the design matrices for their respective terms.

Environmental and block effects were treated as fixed due to the limited number of factor levels (only three environments and two or three blocks per environment in each dataset), which were insufficient for accurate variance component estimation and shrinkage control if treated as random (Gomes 2022).

#### Unweighted two-stage model

For this model type, the analysis was split into two steps, significantly increasing computational speed at the expense of some statistical power. First, within each environment *j*, we fit the following first-stage model:

4 $$\begin{aligned} \boldsymbol{y_j} = W_j\boldsymbol{p_j} + X_j\boldsymbol{\beta _j} + \boldsymbol{\epsilon } \end{aligned}$$

where $\boldsymbol{y_j}$ is a vector of phenotypes in environment *j*, $\boldsymbol{\beta _j}$ is a vector of fixed genotypic effects with the design matrix $X_j$, and $\boldsymbol{\epsilon } \sim N({\boldsymbol{0}}, I\sigma ^2_\epsilon )$ is a vector of independent, identically distributed (i.i.d.) residuals. The term $W_j\boldsymbol{p_j}$ represents a spatial effect similar to that in Eq. 3. The best linear unbiased estimators (BLUEs) for the genotypic effects of each environment were then merged into a single vector ($\boldsymbol{{\hat{\beta }}_{all}}$) by stacking them:

5 $$\begin{aligned} \boldsymbol{{\hat{\beta }}_{all}} = \begin{pmatrix} \boldsymbol{{\hat{\beta }}_{1}} \\ \boldsymbol{{\hat{\beta }}_{2}} \\ \boldsymbol{{\hat{\beta }}_{3}} \end{pmatrix} \end{aligned}$$

In the second stage model, the genotypic effects were further divided into additive and non-additive components:

6 $$\begin{aligned} \boldsymbol{{\hat{\beta }}_{all}} = X_e{\boldsymbol{e}} - ({\boldsymbol{F}}b) + Z_a{\boldsymbol{a}} + Z_g {\boldsymbol{g}} + \boldsymbol{\epsilon } \end{aligned}$$

where all terms follow the same nomenclature as in Eq. 3.

#### Fully efficient two-stage model

Fully efficient models combine the computational efficiency of the UNW model with most of the statistical power of the SS model (Piepho et al. 2012). The first stage of the FE model is the same as that of the UNW model (Eq. 4), where the $\boldsymbol{{\hat{\beta }}_{all}}$ BLUEs are obtained as in Eq. 5. Furthermore, their variance (EEV) is also extracted. The term EEV is calculated from the inverse of the coefficient matrix of the Henderson equations, as explained in Fernández-González and Isidro y Sánchez, J. (2025). The EEV matrices from each environment are merged using a direct sum:

7 $$\begin{aligned} EEV_{all} = \begin{pmatrix} EEV_{1} & \quad 0 & \quad 0 \\ 0 & \quad EEV_{2} & \quad 0 \\ 0 & \quad 0 & \quad & \quad EEV_{3} \\ \end{pmatrix} \end{aligned}$$

$\boldsymbol{{\hat{\beta }}_{all}}$ and $EEV_{all}$ are then used as inputs for the second stage model:

8 $$\begin{aligned} \boldsymbol{{\hat{\beta }}_{all}} = X_e{\boldsymbol{e}} - ({\boldsymbol{F}}b) + Z_a{\boldsymbol{a}} + Z_g {\boldsymbol{g}} + {\boldsymbol{s}} + \boldsymbol{\epsilon } \end{aligned}$$

where all terms are similar to those in Eq. 6. Here, ${\boldsymbol{s}} \sim N({\boldsymbol{0}}, EEV_{all})$ is a random effect that accounts for estimation errors from the first stage. In classical FE models, $EEV_{all}$ is included as the covariance matrix of the residuals (Piepho et al. 2012). However, this would prevent the residuals from capturing other unaccounted sources of variability, such as genotype by environment interactions, as shown in Fernández-González and Isidro y Sánchez, J. (2025). Including $EEV_{all}$ as a random effect resolves this issue, as demonstrated by Endelman (2023) and Fernández-González and Isidro y Sánchez, J. (2025).

Although FE models are mathematically equivalent to SS when variance components are known, this condition is rarely met in practice (Piepho et al. 2012; Damesa et al. 2017). Variance components must instead be estimated, and because FE models rely on single-environment data in the first stage, this estimation may be less reliable and lead to modest losses in prediction accuracy (Piepho et al. 2012; Damesa et al. 2017, 2019; Fernández-González and Isidro y Sánchez, J. 2025). Thus, SS is expected to be slightly more accurate, while FE models remain faster and more flexible (Appendix 1).

## Results

**Table 2:** Accuracy values obtained for the three datasets. All values correspond to the average across simulation runs plus-minus the standard error of the mean. Within each dataset, ten different scenarios corresponding to different field sizes are displayed. For each one, we included the accuracy on the test set (TS) or training set (TRS). The accuracy can correspond to the classical statistical analysis, i.e., randomized complete block design (RCBD) with unweighted two-stage model (UNW) with block effects; or to a more complex statistical analysis with optimized, partially replicated designs (p-rep_O) with a single-stage model (SS) with 2D p-splines for spatial analysis. Finally, the values under the “Additive” columns correspond to the accuracy of prediction of purely additive breeding values while the values under the “Genotypic” columns correspond to the accuracy of prediction of the additive plus non-additive genetic effects

		TS				TRS			
		RCBD, UNW, Block		p-rep_O, SS, Splines		RCBD, UNW, Block		p-rep_O, SS, Splines	
Dataset	Scenario	Additive	Genotypic	Additive	Genotypic	Additive	Genotypic	Additive	Genotypic
Maize	2 Blocks x 25 Plots	0.44±0.01	0.30±0.01	0.63±0.04	0.49±0.11	0.67±0.01	0.59±0.01	0.75±0.03	0.58±0.19
	2 Blocks x 50 Plots	0.59±0.01	0.50±0.01	0.69±0.01	0.59±0.01	0.70±0.01	0.74±0.01	0.75±0.01	0.75±0.01
	2 Blocks x 100 Plots	0.71±0.00	0.63±0.00	0.77±0.00	0.69±0.00	0.75±0.00	0.77±0.00	0.78±0.00	0.71±0.00
	2 Blocks x 150 Plots	0.76±0.00	0.68±0.00	0.80±0.00	0.70±0.00	0.78±0.00	0.71±0.00	0.80±0.00	0.76±0.00
	2 Blocks x 200 Plots	0.79±0.00	0.71±0.00	0.81±0.00	0.72±0.00	0.80±0.00	0.71±0.00	0.82±0.00	0.76±0.00
	3 Blocks x 25 Plots	0.45±0.01	0.30±0.01	0.59±0.01	0.48±0.01	0.67±0.01	0.59±0.01	0.69±0.01	0.69±0.01
	3 Blocks x 50 Plots	0.60±0.01	0.51±0.01	0.72±0.01	0.62±0.01	0.71±0.01	0.76±0.01	0.74±0.00	0.73±0.00
	3 Blocks x 100 Plots	0.71±0.00	0.62±0.01	0.78±0.00	0.70±0.00	0.75±0.00	0.77±0.00	0.79±0.00	0.72±0.00
	3 Blocks x 150 Plots	0.77±0.00	0.68±0.00	0.81±0.00	0.71±0.00	0.78±0.00	0.71±0.00	0.81±0.00	0.76±0.00
	3 Blocks x 200 Plots	0.78±0.00	0.71±0.00	0.82±0.00	0.76±0.00	0.80±0.00	0.70±0.00	0.82±0.00	0.74±0.00
Sunflower	2 Blocks x 25 Plots	0.39±0.01	0.31±0.01	0.39±0.04	0.47±0.05	0.66±0.01	0.68±0.02	0.62±0.04	0.78±0.03
	2 Blocks x 50 Plots	0.47±0.01	0.46±0.01	0.52±0.02	0.60±0.02	0.67±0.01	0.79±0.01	0.66±0.02	0.76±0.02
	2 Blocks x 100 Plots	0.52±0.01	0.61±0.01	0.56±0.01	0.66±0.01	0.68±0.01	0.76±0.01	0.67±0.01	0.76±0.01
	2 Blocks x 150 Plots	0.55±0.01	0.65±0.01	0.58±0.01	0.67±0.01	0.68±0.01	0.77±0.01	0.67±0.01	0.78±0.01
	2 Blocks x 200 Plots	0.57±0.01	0.66±0.01	0.58±0.01	0.67±0.01	0.68±0.01	0.77±0.01	0.65±0.01	0.74±0.01
	3 Blocks x 25 Plots	0.38±0.01	0.33±0.01	0.47±0.02	0.47±0.02	0.64±0.02	0.70±0.02	0.65±0.01	0.67±0.02
	3 Blocks x 50 Plots	0.47±0.01	0.47±0.01	0.54±0.01	0.61±0.01	0.67±0.01	0.80±0.01	0.66±0.01	0.74±0.01
	3 Blocks x 100 Plots	0.53±0.01	0.61±0.01	0.57±0.01	0.67±0.01	0.68±0.01	0.77±0.01	0.66±0.01	0.75±0.01
	3 Blocks x 150 Plots	0.56±0.01	0.66±0.01	0.58±0.01	0.67±0.01	0.69±0.01	0.78±0.01	0.64±0.01	0.74±0.01
	3 Blocks x 200 Plots	0.57±0.01	0.66±0.01	0.59±0.01	0.66±0.01	0.68±0.01	0.78±0.01	0.64±0.01	0.72±0.01
Oat	2 Blocks x 25 Plots	0.61±0.02	0.76±0.01	0.64±0.01	0.79±0.01	0.73±0.01	0.93±0.00	0.74±0.01	0.92±0.00
	2 Blocks x 50 Plots	0.59±0.02	0.72±0.01	0.58±0.03	0.76±0.01	0.73±0.01	0.92±0.00	0.70±0.03	0.92±0.01
	2 Blocks x 100 Plots	0.61±0.02	0.76±0.01	0.64±0.01	0.79±0.01	0.73±0.01	0.93±0.00	0.74±0.01	0.92±0.01
	2 Blocks x 150 Plots	0.62±0.02	0.78±0.01	0.65±0.01	0.81±0.01	0.73±0.01	0.93±0.00	0.74±0.01	0.93±0.00
	2 Blocks x 200 Plots	0.64±0.01	0.80±0.01	0.67±0.01	0.83±0.00	0.74±0.01	0.93±0.00	0.75±0.01	0.93±0.00
	3 Blocks x 25 Plots	0.57±0.02	0.66±0.01	0.57±0.03	0.72±0.01	0.72±0.02	0.91±0.01	0.68±0.02	0.89±0.01
	3 Blocks x 50 Plots	0.60±0.02	0.72±0.01	0.62±0.02	0.77±0.01	0.74±0.01	0.92±0.00	0.72±0.01	0.91±0.00
	3 Blocks x 100 Plots	0.61±0.02	0.76±0.01	0.65±0.01	0.81±0.01	0.74±0.01	0.93±0.00	0.73±0.01	0.92±0.00
	3 Blocks x 150 Plots	0.63±0.01	0.79±0.01	0.67±0.01	0.83±0.00	0.74±0.01	0.93±0.00	0.74±0.01	0.92±0.00
	3 Blocks x 200 Plots	0.65±0.01	0.80±0.01	0.68±0.01	0.85±0.00	0.75±0.01	0.93±0.00	0.75±0.01	0.93±0.00

Table 2 summarizes prediction accuracy across scenarios under both the classical experimental design and modeling strategy and the approach proposed in this study. Overall, predictive accuracy was high, reaching a maximum of 0.93 for genotypic values in the oat dataset. As expected, accuracy broadly reflected trait heritability (Table 1), with the highest values observed in oat, followed by maize and sunflower. Accuracy for the unobserved testing set (TS) was consistently lower than for the training set (TRS), reflecting the greater challenge of predicting unphenotyped material. Increasing field size, and therefore TRS size, improved prediction accuracy in the TS, whereas its effect on within-TRS accuracy was more limited. These trends are consistent with theoretical expectations, as higher heritability and larger training populations generally increase predictive ability.

For genotypic values (additive + non-additive), accuracy was consistently higher than for additive values in the sunflower and oat datasets. However, this trend was reversed in the maize dataset. Across all scenarios, the p-rep_O design combined with the SS model and 2D P-splines consistently yielded higher prediction accuracy on an unobserved TS than the RCBD design with the UNW model and block effects. Conversely, within the TRS, the accuracy differences between these two approaches were minimal.

A comparison between the scenarios of 2 blocks with 150 plots and 3 blocks with 100 plots is particularly insightful, as both configurations contain the same number of plots. However, the 2-block scenario includes a larger number of unique genotypes with fewer replications, whereas the 3-block scenario tests fewer genotypes but with increased replication. Generally, both scenarios presented similar accuracy for within-TRS predictions, but the 2 blocks × 150 plots configuration provided slightly higher accuracy for predicting an unobserved TS.

Scenarios with 100 plots or fewer (2 blocks × 25 plots, 2 blocks × 50 plots, and 3 blocks × 25 plots) showed convergence problems when fitting models with 2D P-splines. Although some repetitions converged successfully, most failed during model calibration, indicating that small field trials may provide insufficient degrees of freedom to support complex spatial modeling, especially under the p-rep_O design. These convergence failures reduced the available sample size and increased the standard error of the mean, in the affected scenarios reported in Table 2. Consequently, subsequent analyses excluded results from models using 2D P-splines in these scenarios, as their convergence instability makes them unsuitable for practical breeding applications.

### Sources of accuracy gain

Table 2 shows that the classical analysis, based on RCBD design with a UNW model and block effects, generally performs worse than the more complex analyses, such as the p-rep_O design with an SS model and 2D P-splines. While both approaches yield similar accuracy for within-TRS predictions, the latter demonstrated substantially higher accuracy for TS predictions. However, these two types of analyses differ significantly in their methodologies, and a simple presentation of overall accuracy, as in Table 2, does not disentangle the contributions of each factor. Figures 2 through 4 facilitate a more detailed exploration by isolating the influence of individual factors and examining their interactions.

We will illustrate the interpretation of these figures using the section for small fields in Fig. 2 as an example. As a baseline, the accuracy of the classical analysis (RCBD design with a UNW model and block effects) was recorded. This baseline was then compared to the accuracy of the p-rep_R design while keeping all other factors constant, revealing a 14.62% improvement in accuracy for the p-rep_R design.

The effect of model type on accuracy was evaluated for the p-rep_R design. Transitioning to the SS model increased accuracy by 1.28%, while using the FE model resulted in a 0.83% improvement over the UNW model. Subsequently, replacing the randomized p-rep_R design with the optimized p-rep_O design enhanced prediction accuracy by 1.87% to 2.54%, depending on the model type employed.

Finally, the potential impact of substituting block effects in the models with 2D P-splines was considered. However, as noted earlier, this substitution was not feasible for small field trials due to convergence issues.

The total accuracy gain represents the cumulative improvement achieved by combining all individual changes. It is important to note that this total gain is not a simple sum of the individual contributions. Instead, accuracy gains are multiplicative, and the compounding effects of sequential improvements must be accounted for.

**Fig. 3:**
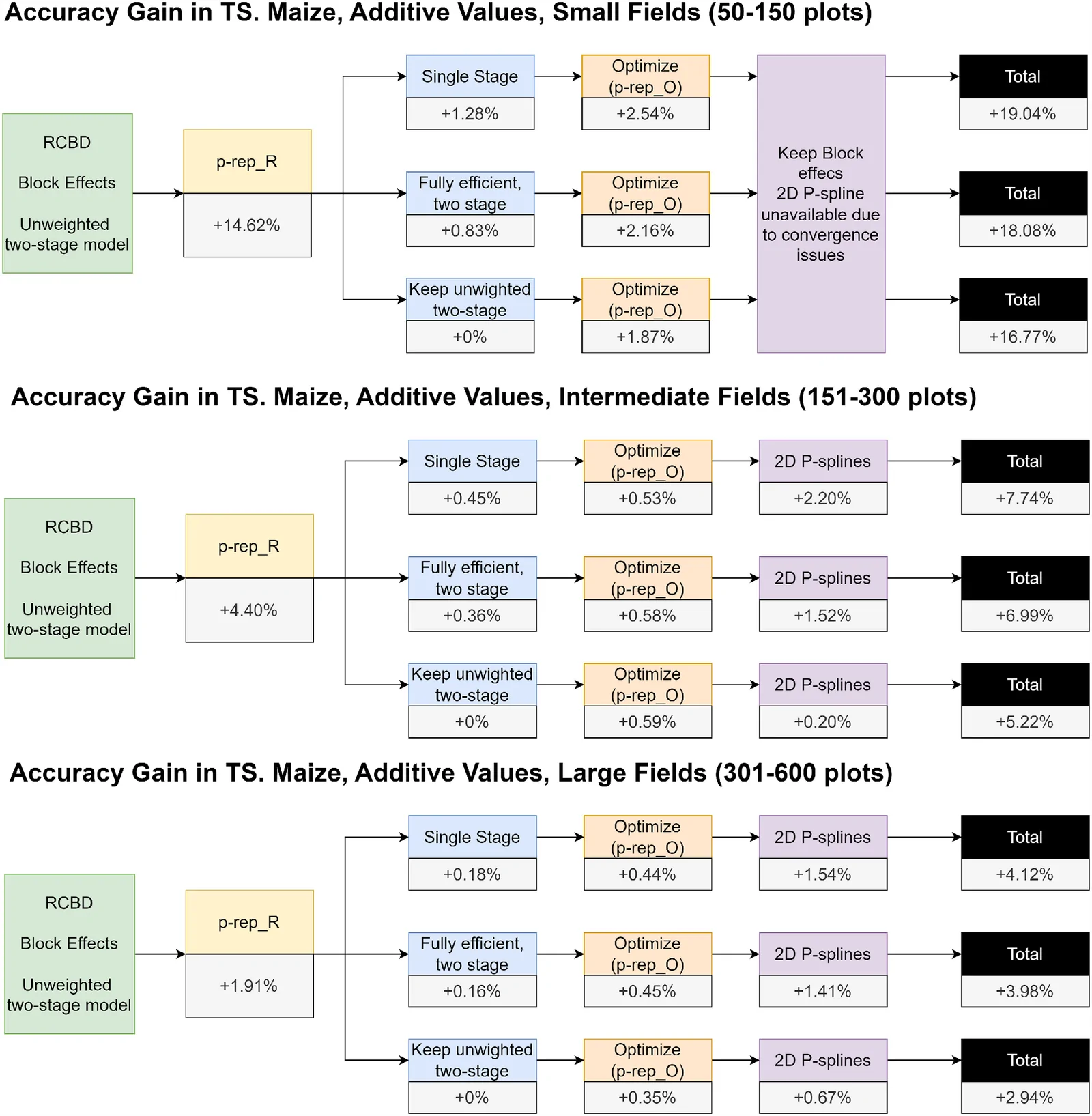
Changes in test set (TS) prediction accuracy for additive genetic values in the Maize dataset as increasingly complex experimental designs and modeling are sequentially implemented

**Fig. 4:**
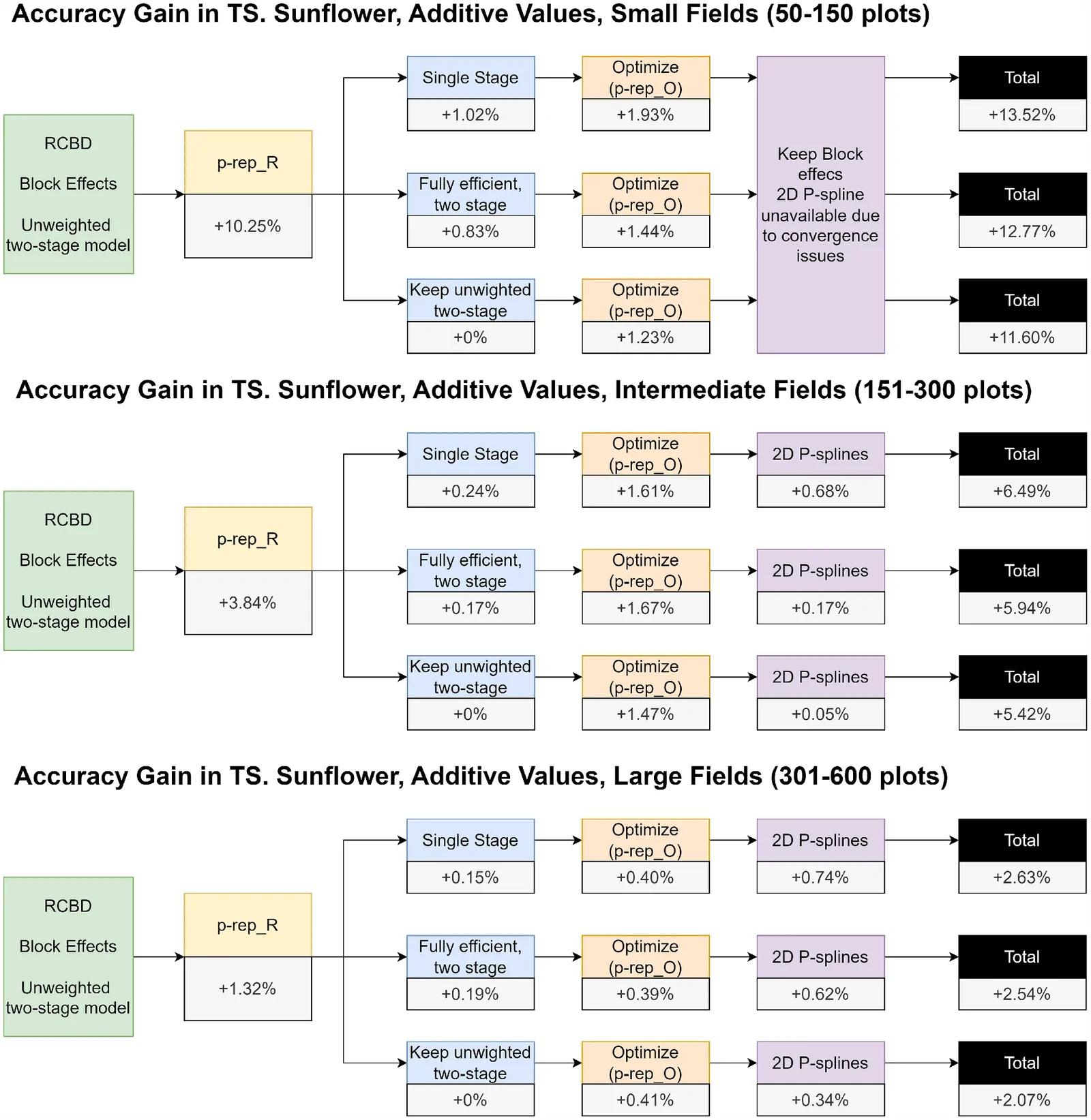
Changes in test set (TS) prediction accuracy for additive genetic values in the Sunflower dataset as increasingly complex experimental designs and modeling are sequentially implemented

**Fig. 5:**
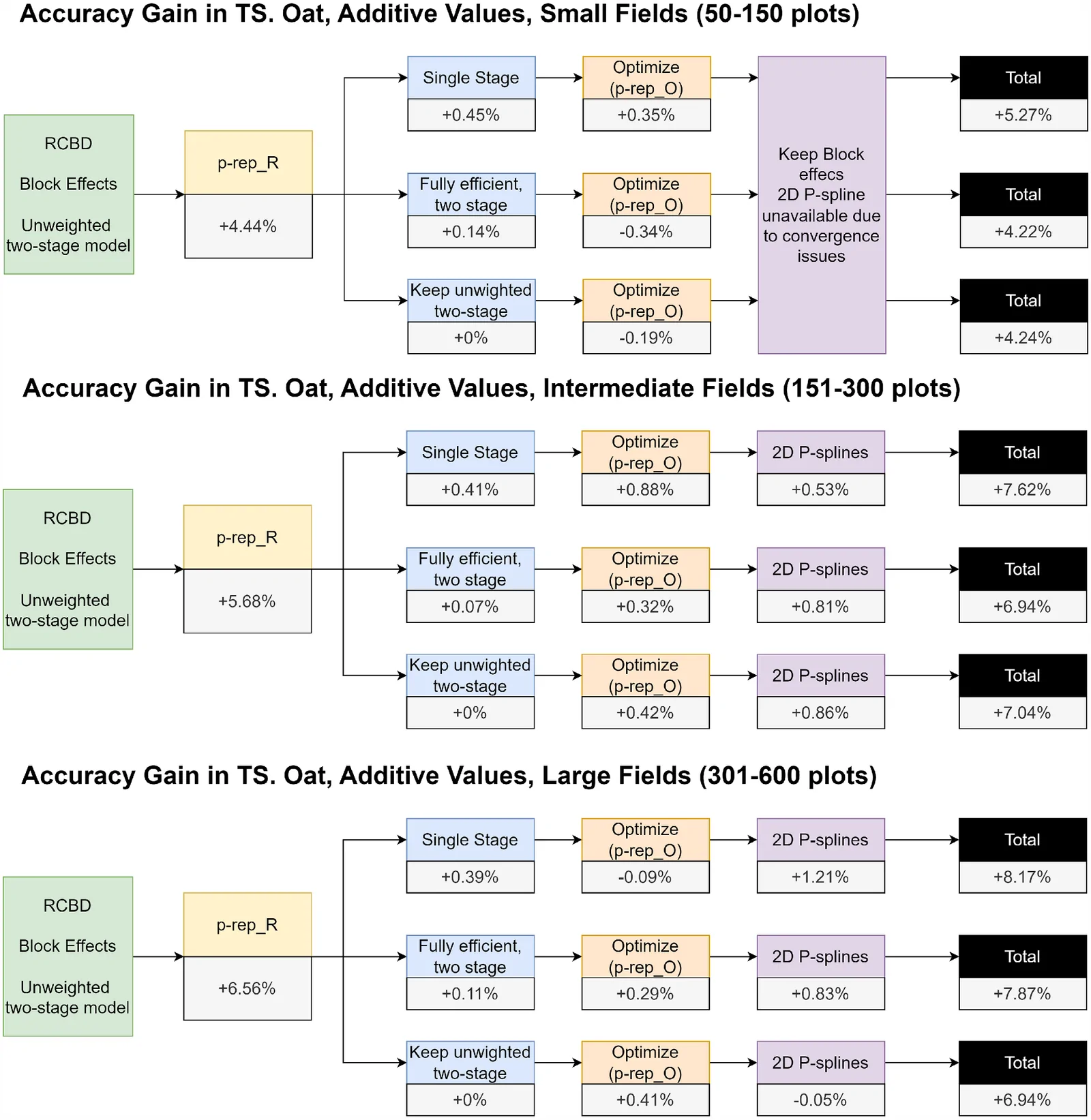
Changes in test set (TS) prediction accuracy for additive genetic values in the Oat dataset as increasingly complex experimental designs and modeling are sequentially implemented

In all cases, transitioning from an RCBD design to a p-rep_R design significantly improved TS accuracy. For hybrid crops (Figs. 2, 3), this improvement was particularly pronounced in small field trials, with accuracy gains exceeding 10%. However, the magnitude of this gain decreased rapidly as the field size increased, falling below 2% for the largest trials. In contrast, for the self-pollinated oat dataset (Fig. 4), the accuracy gain from switching to the p-rep_R design was more modest in small field trials (4.44%) but increased with larger field trials, reaching a maximum of 6.56% for the largest trials.

Regarding the model types, SS models consistently outperformed FE models, which, in turn, performed better than UNW models across all datasets. The accuracy gain from using the SS model was generally more pronounced in small field trials (around 1% for the SS model) compared to larger trials, where gains ranged between 0.15% and 0.39%.

Optimizing the experimental design by transitioning from p-rep_R to p-rep_O resulted in accuracy gains of 1.23% to 2.54% for small field trials in hybrid crops (Figs. 2, 3). However, the impact of optimization diminished with larger trials, where gains were approximately 0.40%. In the oat dataset (Fig. 4), the accuracy improvement from optimization was smaller, ranging from −0.34% to 0.88%, with no clear trend relative to field size. In some scenarios, the benefits of optimization were amplified when combined with SS or FE models, but this interaction was inconsistent across datasets and field sizes.

Finally, the 2D P-splines were unavailable for small field trials due to convergence issues, but they provided substantial gains in other scenarios, particularly when combined with SS or FE models. For the SS model, the accuracy gain from 2D P-splines ranged between 0.53 and 2.20% across all scenarios; for FE models, gains ranged from 0.17 to 1.52%, and for UNW models, gains were minimal and ranged from −0.05 to 0.86%. These results highlight that UNW models were barely able to leverage gains from 2D P-splines, whereas FE and especially SS models capitalized on them effectively.

The overall total gains from all factors were considerable across all scenarios. Gains were particularly remarkable in small field trials for hybrid datasets (Figs. 2, 3), with total accuracy improvements ranging from 10 to 20%. Conversely, as the field size increased, total gains diminished, reaching as low as 2.07% in the sunflower dataset for large field trials with the UNW model. In the oat dataset (Fig. 4), total gains were much more stable across field sizes, ranging from 4.22 to 8.17%, with greater improvements observed for larger field sizes.

All results discussed above apply exclusively to additive values. Supplementary Figures S1 - S3 show the results for genotypic values (additive plus non-additive). While the overall trends were largely consistent with those described above, we noted several key differences. For hybrid crops (Figures S1 and S2), the accuracy gain for genotypic values in small field trials was significantly larger than for additive values, reaching approximately 30%. This increase was primarily driven by the substantial impact of transitioning from RCBD to p-rep designs. For larger field sizes, accuracy gains for genotypic values were similar to or slightly smaller than those observed for additive values. In the oat dataset (Figure S3), accuracy gains for genotypic values tended to be slightly lower than those reported for additive values in Fig. 4, ranging from 5.03 to 6.94%.

We also evaluated the accuracy of estimating additive values (Figures S4–S6) and genotypic values (Figures S7 through S9) within the TRS. In general, within-TRS accuracy tended to decrease when transitioning from the fully replicated RCBD design to p-rep designs, with changes ranging from an 8.18% reduction to a 3.41% increase. The transition to p-rep designs had a more pronounced negative impact on the estimation of genotypic values within the TRS compared to additive values. The most substantial reductions were observed in the sunflower dataset, followed by the oat dataset. In contrast, the effect in maize was inconsistent, with some scenarios even showing improvements in accuracy.

All other factors (transitioning to SS or FE models, optimizing the design, and using 2D P-splines) generally had a similar impact on within-TRS accuracy as observed for TS accuracies. These adjustments partially offset the accuracy reduction caused by moving from the RCBD to p-rep designs within the TRS. However, they were often insufficient to surpass the performance of the classical analysis (RCBD with the UNW model and block effects). Total within-TRS accuracy varied significantly from a 7.27% decrease (in the sunflower dataset for small field sizes; Figure S8) to a 6.82% increase (in the maize dataset for large field sizes; Figure S7).

## Discussion

In this work, we used simulations to assess how different modifications to experimental designs and modeling strategies affect prediction accuracy. This approach allowed us to isolate the impact of each improvement, identify interactions among factors, and estimate the overall effect on prediction accuracy and selection response.

### Trade-off between training set size and replication

Increasing the TRS size is known to improve the prediction accuracy of genetic effects (Clark et al. 2012; Rincent et al. 2012; Sánchez et al. 2015; Rincent et al. 2017; Guo et al. 2019; Akdemir and Isidro-Sánchez 2019; Fernández-González et al. 2023), while strong replication enhances the reliability of these estimates (Fisher 1926; Casler 2015; Zystro et al. 2018). However, simultaneously expanding the TRS size and replication would require prohibitively large field trials. Thus, with a limited number of available plots, finding the optimal balance between TRS size and replication is essential.

Figures 2 through 4 demonstrate that, to predict an unobserved TS, p-rep designs (low replication, large TRS size) consistently outperformed RCBD (high replication, smaller TRS size). This advantage was particularly pronounced in hybrid crops (Figs. 2, 3) for small field trials. This trend aligns with prior findings (Clark et al. 2012; Rincent et al. 2012; Sánchez et al. 2015; Rincent et al. 2017; Guo et al. 2019; Akdemir and Isidro-Sánchez 2019; Fernández-González et al. 2023), which reported that prediction accuracy increases sharply at first with TRS size but plateaus as the TRS size continues to grow. Thus, it makes sense that large field trials, which have an inherently large TRS size with any design, benefit less from further increasing it by moving to p-rep designs. For maize and sunflower, the accuracy gain from p-rep designs was around 2% for the largest field trials, suggesting that the TRS size in RCBD trials was sufficient to include a representative sample of the overall population.

In contrast, for the self-pollinated oat dataset, the accuracy gain from p-rep designs was more pronounced in larger field trials (Figs. 4), likely due to the strong epistatic effects present in the simulations. An extremely large TRS would be needed to properly discriminate additive from epistatic effects (Hill et al. 2008), suggesting that even the largest RCBD TRS was insufficient, leaving considerable potential for improvement through further increases in TRS.

This observation is consistent with Table 2, where the accuracy of genotypic values in the oat dataset was high, while the accuracy of additive effects alone was lower. This implies that while predicting the combined additive and epistatic effects was feasible, isolating the additive effects presented challenges, likely due to the difficulty in fully discriminating between additive and epistatic contributions (Hill et al. 2008; Muñoz et al. 2014).

When estimating additive and genotypic values within the TRS (Supplementary Figures S4 through S9), the RCBD design generally outperformed p-rep, especially in the sunflower and oat datasets. This is because increasing the TRS size is less critical for within-TRS estimation, as the genetic diversity of the genotypes being estimated is already fully represented in the observed genotypes. Instead, the increased replication in the RCBD directly enhances accuracy in this scenario.

The larger TRS size in p-rep designs may still be beneficial for discriminating between additive and non-additive genetic effects (Hill et al. 2008), which could explain why the p-rep design often performed better than the RCBD in the maize dataset. Additionally, while p-rep designs typically provide lower accuracy, they allow for the evaluation of a greater number of unique genotypes. This expands genetic variance within the TRS and increases selection intensity, which can compensate for the reduction in accuracy, as seen from the breeder’s equation: $R_t = \frac{r i \sigma _a}{t}$, where $R_t$ is the response from selection, *r* is accuracy, *i* is the intensity of selection, $\sigma _a$ is the additive standard deviation in the population, and *t* is the generation interval (Lush 1943). For example, in small field trials in sunflower, the worst performance of p-rep designs for within-TRS genotypic value prediction occurred with p-rep_R, which was 8.18% less accurate than RCBD (Figure S8). In a field with 2 blocks and fifty plots per block, RCBD tests fifty genotypes, while p-rep evaluates ninety. Selecting the 10 best individuals corresponds to a selection intensity of i=1.40 for RCBD and i=1.70 for p-rep, representing a 21.77% increase. Consequently, the response from selection with p-rep would be greater than RCBD by a factor of $(1+\frac{21.77}{100})(1-\frac{8.18}{100}) = 1.1181$, or an 11.81% increase. This demonstrates that the higher selection intensity compensates for the reduced accuracy in p-rep designs.

Thus, p-rep designs are highly recommended for early field trials where evaluating a large number of genotypes is advantageous. For advanced trials, where the goal is to assess a smaller pool of genotypes with greater reliability, within-TRS accuracy becomes more critical, and RCBD remains the preferred choice (Zystro et al. 2018).

Finally, as noted by Zystro et al. (2018), p-rep designs are generally better suited for traits with high heritability, where high replication is less critical due to minimal noise from non-genetic effects. However, our findings demonstrate that the p-rep design can also be effective for traits with very low heritability. We focused on simulating yield, a trait known for its low heritability (Table 1), which makes our scenario a worst-case scenario for p-rep designs. Despite this, p-rep designs performed remarkably well.

This success is likely due to the advanced statistical methods employed. The use of the genomic relationship matrix enables information sharing among genotypes, which can mimic the effects of increased replication (Endelman et al. 2014). Additionally, while replication is traditionally essential for robust spatial analysis, 2D P-splines provide high-resolution spatial modeling with relatively few degrees of freedom (Eilers et al. 2015), effectively reducing the need for extensive replication. Although we encountered convergence issues with 2D P-splines in very small field trials (fewer than 150 plots), they consistently delivered strong performance in most scenarios.

These results highlight that, with appropriate statistical tools, p-rep designs can be successfully applied even under the challenging conditions of low heritability, expanding their utility beyond traits with inherently high genetic predictability.

### Modeling and spatial analysis

As shown in Figs. 2 through 4, SS was the best performing model, followed by FE and UNW, which is consistent with previous findings (Piepho et al. 2012; Fernández-González and Isidro y Sánchez, J. 2025). However, it is important to note that SS can be substantially slower and less versatile than two-stage analysis (more details in Appendix 1), which can make it unfeasible in some scenarios. FE performed almost as well as SS, making it a very appealing alternative when SS is not viable.

2D P-splines combine high resolution with strong performance (Rodríguez-Álvarez et al. 2017; Lee et al. 2013; Eilers et al. 2015). In this study, we found that they could cause convergence issues in very small fields. This limitation is not critical, as small fields result in smaller, more homogeneous blocks, making it reasonable to assume no within-block variation and to use block effects instead (Zystro et al. 2018). For larger fields, which have more heterogeneous blocks, 2D P-splines outperform block effects, as seen in Figs. 2 - 4.

An interesting interaction was observed between 2D P-splines and model types. Accuracy gains from 2D P-splines were modest in UNW models but significantly larger for FE and SS models. This difference likely arises because UNW models do not simultaneously account for spatial analysis (first stage) and genomic relationships (second stage). In contrast, SS models incorporate both within a single-stage, and FE models link both through the EEV matrix, achieving a similar effect. Indeed, FE models are mathematically equivalent to SS when the variance components are known (Piepho et al. 2012). In SS and FE models, genomic relationships enable genotypes to borrow information from one another, mimicking the effect of increased replication (Endelman et al. 2014), which enhances plot effect estimation. Consequently, FE and SS models are better able to capitalize on the benefits of 2D P-splines compared to UNW models.

Regarding computational efficiency, 2D P-splines significantly increase computational time in SS models but add negligible costs in UNW and FE models, which is consistent with the findings in Fernández-González and Isidro y Sánchez, J. (2025). Thus, for large field trials, the FE models offer the best balance of speed, performance, the ability to leverage gains from 2D P-splines, and versatility. FE models also allow for different spatial analysis across individual locations or the inclusion of specific covariates in separate experiments, tasks that are more challenging to implement with SS models.

### Impact of experimental design optimization

In this study, we introduced the CDmean criterion for optimizing field trials, offering better computational efficiency than existing alternatives (Cullis et al. 2006; Feoktistov et al. 2017) and supporting targeted optimization (details in Appendix 5). As shown in Figs. 2 through 4 and S1 through S9, this approach significantly increased accuracy on average without imposing substantial additional costs on breeding programs. We anticipated that optimization would perform best when paired with SS or FE models, as these models simultaneously account for genomic relationships and the spatial distribution of genotypes. However, this expectation was not consistently met across all scenarios.

**Table 3:** Accuracy gain from experimental design optimization in all datasets. The displayed values are the average across all scenarios and correspond to the gain from optimization in partially replicated designs (p-rep_O; optimized vs p-rep_R; randomized) or fully replicated designs (OCBD; optimized vs RCBD; randomized) when making predictions on the training set (TRS) or test set (TS)

	p-rep_O vs p-rep_R		OCBD vs RCBD	
	TRS	TS	TRS	TS
Maize	0.44%	0.51%	0.07%	0.45%
Sunflower	0.46%	0.95%	0.00%	0.11%
Oat	0.07%	0.37%	−0.05%	−0.11%

A summary of the optimization performance is provided in Table 3. Optimization was more effective in p-rep designs than in fully replicated ones. This may be because optimizing the placement of the few replicated checks in a p-rep design allows for better control of spatial variability. Additionally, in fully replicated designs, only the spatial distribution of genotypes can be optimized, whereas, in p-rep designs, both the distribution and the selection of genotypes as checks are optimized, increasing the potential benefits. Lastly, optimization performed substantially better in hybrid crops (maize and sunflower) than in the self-pollinated oat dataset. This discrepancy may be due to differences in genetic architecture between these crops or the strong population structure in the oat dataset. Further research is needed to investigate these factors in greater detail.

### Compounding effect of accuracy gain

Many factors that individually contribute modestly to accuracy demonstrate compounding effects when combined, resulting in substantial improvements. As an example, we use the intermediate field size for maize in Fig. 2. In this scenario, transitioning from the RCBD to the p-rep design increased accuracy by 4.40% with no additional cost, as the same number of plots was used. Regarding model types, while SS provided the highest accuracy, its computational cost is often prohibitive. The FE model, however, achieved a 0.36% improvement over UNW at a similar computational cost. Optimization of the experimental design added a further 0.58% accuracy gain, with minimal cost (a large field trial can be optimized within a few hours on a single core). Finally, replacing block effects with 2D P-splines, which incur negligible costs, improved accuracy by 1.52%.

The total gain in this scenario was calculated as $1.0440\cdot 1.0036 \cdot 1.0058 \cdot 1.0152 = 1.0699$, representing a 6.99% improvement. These gains compound multiplicatively rather than additively, underscoring the importance of integrating as many improvements as possible to maximize compounding benefits.

Additionally, there is a second level of compounding through selection cycles. Response from selection is directly proportional to accuracy; thus, a 6.99% accuracy gain translates to a 6.99% greater response from selection per cycle. Over multiple selection cycles, this gain compounds further. For instance, after five cycles, genetic gain would be greater by a factor of $1.0699^5 = 1.4019$, meaning the response from selection would be 40.19% higher with the proposed approach (p-rep_O with the FE model and 2D P-splines) compared to the classical analysis (RCBD with the UNW model and block effects).

### Conclusions

This study highlights the advantages of reducing replication to increase the number of genotypes tested, particularly when advanced statistical analysis is used to mitigate the drawbacks of lower replication.

P-rep designs are generally recommended, as they enable the construction of a more powerful TRS, resulting in higher accuracy for predicting an unobserved TS. While within-TRS accuracy is often lower in p-rep designs compared to RCBD, the larger number of evaluated genotypes increases selection intensity, leading to an overall improvement from selection. Additionally, the optimization of the experimental design provides small but meaningful accuracy improvements. The significance of these incremental gains lies in their compounding effects, both within a single selection cycle and across multiple cycles, ultimately driving substantial long-term genetic gain.

In terms of modeling strategy, 2D P-splines consistently outperformed block effects, except in small field trials (fewer than 150 plots), where convergence issues may arise. To fully capitalize on the benefits of spatial analysis, FE or SS models are preferred over UNW. SS models deliver the highest performance, but they are slower and less versatile than FE. Thus, we recommend using SS whenever it is feasible, and FE otherwise, such as for very large multi-environment trials or when different spatial analysis or covariates are needed for each field.

## Supplementary Information

Below is the link to the electronic supplementary material.

Supplementary file 1 (pdf 6227 KB)

## Figures and Tables

**Fig. 6:**
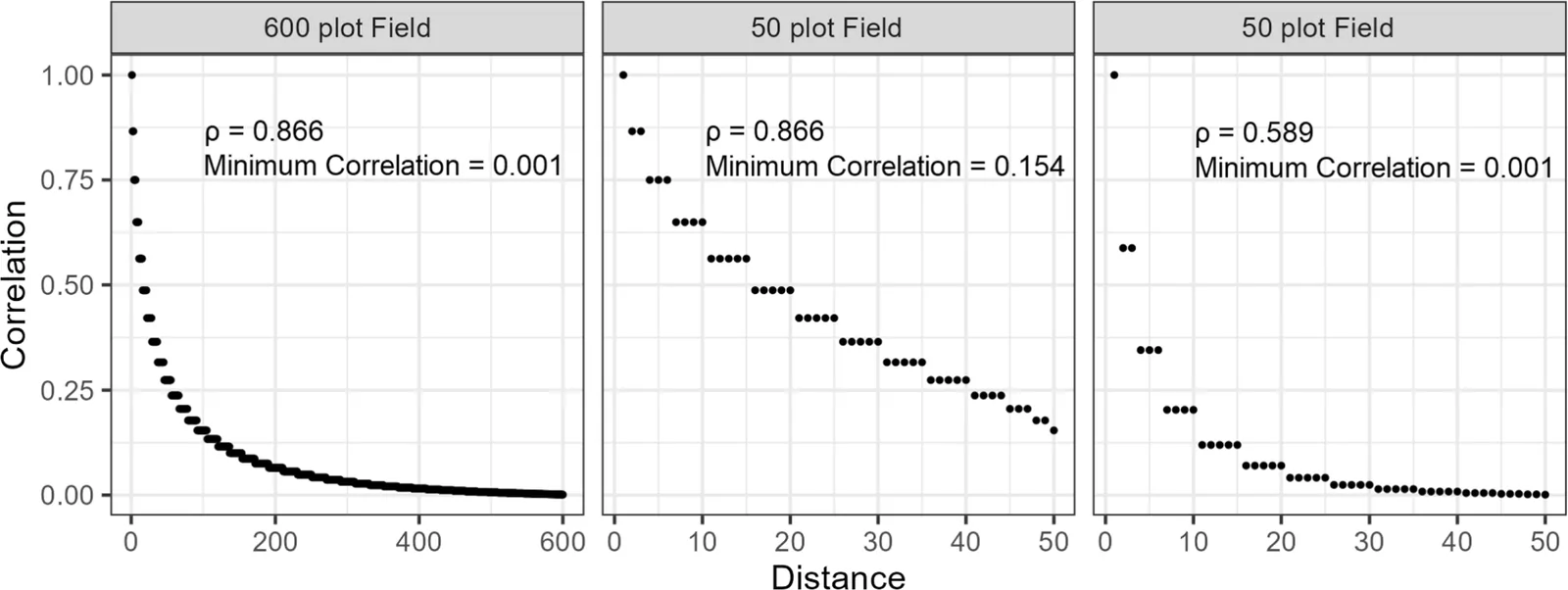
Correlation between plots in a large 600-plot field compared to a small 50-plot field. The vertical axis represents correlation values and the horizontal axis contains the index numbers of the plots sorted by their distance to the top-left corner of the field. In all cases we assumed that $\rho _{rows} = \rho _{cols} = \rho$

## Data Availability

The Maize (Hirsch et al., 2014) and Oat (Canales et al., 2021) datasets were retrieved from previous publications. The sunflower dataset is a corporate one and is not publicly available.

## Appendix 1: Computational cost and practicality of single-stage, two-stage models, and design optimization

Genomic prediction models (and also CDmean optimization) have cubic time complexity due to the matrix products and inversions they require. Let *P* be the total number of plots in the multi-environment trials, n<P be the number of genotypes tested, and *p* be the number of plots within a single location. For the sake of simplicity in this illustrative example, we will assume that all environments have the same number of plots (*p*). Let *e* be the number of environments, such that P=e∗p. Two-stage models fit one model per environment, for a total computational cost in the first stage $O(e*p^3)$ plus $O(n^3)$ in the second stage. In contrast, a single-stage model is $O(P^3) = O(e^3*p^3)$. In a very sparse design within and across environments in which n≈P, the computational cost of single-stage and two-stage models is not too different. In less sparse experimental designs, where n<<P, the total cost of the two-stage strategy is dominated by the cost of the first stage. In this case, $O(e*p^3) < O(e^3*p^3)$, showcasing the computational advantage of two-stage models. The larger the number of environments, the more extreme this difference becomes. For instance, for 3 environments, two-stage models are roughly $3^3p^3/3p^3 = 9$ times faster than single-stage ones. For 10 environments, this difference rises to $10^3p^3/10p^3 = 100$ times faster. This can become an important bottleneck in large breeding programs.

There is another important caveat for the practicality of single-stage models regarding their versatility. In this work, we assumed that all locations presented the same experimental design and thus required the same model to analyze them. In this scenario, it is very simple to build both the single-stage and the two-stage models. However, in practice, it is not uncommon to have different experimental designs and data availability in different environments. Under these circumstances, sometimes different locations require different spatial analysis. For instance, if row-column information is not available for one location, 2D p-splines cannot be fit and an alternative must be used. This is very easy to do with two-stage models, where one model is independently fit for each location. For single-stage models, in contrast, it can be very challenging to correctly program the desired heterogeneous analysis into the software tool. This can become a major roadblock, and it supposes a substantial advantage of two-stage models.

Regarding optimization, it is also $O(e*p^3)$ similarly to two-stage models. However, even if it scales relatively well with dimensions, its base computational cost is much larger than that of the modeling step. Therefore, optimization is typically computationally feasible when each individual field is relatively small (independently of whether there are only a few of many locations), but it becomes unfeasible when individual fields are very large.

## Appendix 2: Variance components determination

The variance components used as parameters in the simulations are presented in Table 4. To maintain constant heritability across different experimental designs, we scaled environmental variances by the harmonic mean of the total number of environments in which each genotype is replicated (*e*) and scaled spatial variances by the harmonic mean of the total number of replications for each genotype (*eb*), following Holland et al. (2003). In the simulations, we set *e* and *eb* to the values they would take in a RCBD, where *e* equals the number of environments and *eb* is the product of the number of environments and the number of blocks per environment.

**Table 4:** Variance components used for the simulation of phenotypes in the different datasets. In all datasets, additive variance has been set to 1 and all other components have been normalized relative to it. *e* refers to the harmonic mean of the number of environments in which each genotype is replicated and *eb* is the harmonic mean of the overall number of times each genotype is replicated

Variance Component	Maize	Sunflower	Oat
Additive	1.000	1.000	1.000
Admixture	0.000	0.000	0.180
Dominance	0.740	0.330	0.000
Epistasis	0.000	0.000	0.260
Environmental	2.050*e*	4.290*e*	0.470*e*
Additive by Environment	0.259*e*	0.316*e*	0.131*e*
Dominance by Environment	0.191*e*	0.104*e*	0.000
Epistasis by Environment	0.000	0.000	0.029*e*
Spatial	0.361*eb*	0.310*eb*	0.09*eb*
Additive by Spatial	0.046*eb*	0.023*eb*	0.025*eb*
Dominance by Spatial	0.034*eb*	0.008*eb*	0.000
Epistasis by Spatial	0.000	0.000	0.005*eb*
Residual	0.22*eb*	0.17*eb*	0.060*eb*

Next, we describe how the variance components were calculated for each dataset. It is important to note that these values are not universal estimates of the variance components for these crops; they will vary depending on the population, environment, experimental design, and other factors. However, they can be considered plausible values broadly representative of the genetic control of crop yield based on a limited set of empirical field trials.

### Maize dataset

Variance components estimated for maize hybrid yield were entirely derived from the literature. According to Hallauer et al. (2010), epistasis in maize hybrids is negligible; therefore, we assumed zero variance for it. We grouped all effects into additive, dominance, overall environmental (including main environmental effects and genotype by environment interaction), and spatial-residual (encompassing all spatial variances and the true error). Their relative values were adjusted to match the variance components and heritabilities reported in the literature (Hallauer et al. 2010; Coutiño-Estrada and Vidal-Martínez 2006; Carvalho et al. 2017; Ferreira Coelho et al. 2021; Yue et al. 2022), with a strong emphasis on Table 5.5 from Hallauer et al. (2010).

To further split the overall environmental and spatial-residual groups into their components, we made several assumptions. For the overall environmental group, most studies only estimate the variance of genotype by environment interactions, not the main environmental variance. However, Coutiño-Estrada and Vidal-Martínez (2006) found that the main environmental variance was roughly five times larger than the genotype by environment interaction, a value we adopted. Additionally, we split the genotype by environment interaction into additive by environment and dominance by environment interactions, assuming the same ratio as between the main additive and dominance effects.

For the spatial-residual group, we used data from Table 4 in Ferreira Coelho et al. (2021), where the residual variance with an autoregressive structure (i.e., spatial variance) was 572230.99, and the residual variance with i.i.d. errors (i.e., spatial plus residual variance) was 862757.39. This gives us the ratio between spatial effects (variance = 572230.99) and the residual (variance = 862757.39-572230.99=290526.4), showing that the spatial variance was roughly twice the residual variance. We further partitioned the spatial variance into main spatial effects and genotype by spatial effects, assuming the same ratios as those between the environmental and genotype by environment effects. This approach reflects the idea that spatial effects represent environmental variation within the field, fundamentally similar to the environmental effects across locations.

### Sunflower dataset

We fitted several models to the sunflower dataset and selected the one with the lowest Akaike Information Criterion (AIC):

$$\begin{aligned} {\boldsymbol{y}} = {\boldsymbol{1}}\mu + Z_a {\boldsymbol{a}} + Z_d {\boldsymbol{d}} + Z_e {\boldsymbol{e}} + Z_{ge} {{\boldsymbol{g}}}{{\boldsymbol{e}}} + Z_p {\boldsymbol{p}} + \boldsymbol{\epsilon } \end{aligned}$$

where ${\boldsymbol{y}}$ is a vector of phenotypic records, μ is a fixed intercept, ${\boldsymbol{a}} \sim N({\boldsymbol{0}}, G\sigma ^2_a)$ is a vector of additive effects, ${\boldsymbol{d}} \sim N({\boldsymbol{0}}, D\sigma ^2_d)$ is a vector of dominance effects. The vector ${\boldsymbol{e}} \sim N({\boldsymbol{0}}, I\sigma ^2_e)$ represents the main environmental effects, ${{\boldsymbol{g}}}{{\boldsymbol{e}}} \sim N({\boldsymbol{0}}, \text {diag}(e) \otimes I)$ captures the genotype by environment interaction, ${\boldsymbol{p}} \sim N({\boldsymbol{0}}, \text {diag}(e) \otimes I)$ represents block effects nested within environments, and $\boldsymbol{\epsilon } \sim N({\boldsymbol{0}}, I\sigma ^2_\epsilon )$ represents the residuals. We chose homogeneous variance of residuals across environments because models with heterogeneous residuals had higher AIC values.

Here, *G* is the VanRaden (2008) additive relationship matrix, *D* is the Vitezica et al. (2013) dominance relationship matrix, *I* is the identity matrix, and diag*(e)* is a diagonal covariance structure among environments. ${\boldsymbol{1}}$ is a vector of ones (design matrix of the intercept), and $Z_a$, $Z_d$, $Z_e$, $Z_{ge}$, and $Z_p$ are the design matrices for their corresponding effects.

The variance components for the simulation were derived from the restricted maximum likelihood estimates of the model when the covariance matrix of the random effects was diagonal. Conversely, the PEV estimates of the variance components were employed for the additive and dominance effects (which have non-diagonal covariance structures), as outlined in Fernández-González et al. (2025). The PEV estimate is calculated by adding the variance of the best linear unbiased predictors to the mean of the diagonal elements of the PEV matrix. Variances for effects with heterogeneous variance across environments (e.g., block effects and genotype by environment interaction) were calculated as weighted averages of the variances in each environment, with weights reflecting the number of observations per environment.

The genotype by environment effects were split into additive by environment and dominance by environment components, using the same ratio as the main additive and dominance variance components. For the spatial term, because block effects do not capture within-block variance, we adjusted the spatial variance to reflect the ratio between spatial and residual variances found in maize by Ferreira Coelho et al. (2021). The spatial variance was further divided into a main effect and its interaction with additive and dominance genotypic effects, following the same ratios as those between the main environmental effects and their genotype interactions.

The variances extracted from the model resulted in very low heritabilities, consistent with some previous studies (Hu et al. 2010; Ahmed et al. 2020), but significantly lower than those of others (Khan 2001). This discrepancy may arise from differences in plant material and experimental design, which can influence heritability estimates.

### Oat dataset

We fitted several models to the oat dataset and selected the one with the lowest Akaike Information Criterion (AIC):

$$\begin{aligned} {\boldsymbol{y}} = X_e \boldsymbol{\beta _{e}} + X_{admix}\boldsymbol{\beta _{admix}} + X_p \boldsymbol{\beta _{p}} + Z_a {\boldsymbol{a}} + Z_{g} {\boldsymbol{g}} + Z_{ge} {{\boldsymbol{g}}}{{\boldsymbol{e}}} + \boldsymbol{\epsilon } \end{aligned}$$

where ${\boldsymbol{y}}$ is a vector of phenotypic records, $\boldsymbol{\beta _{e}}$ is a vector of fixed environmental effects, $\boldsymbol{\beta _{admix}}$ is a vector of fixed regression coefficients for the admixture profile of the genotypes, and $\boldsymbol{\beta _{p}}$ is a vector of fixed block effects nested within the environment. The vector ${\boldsymbol{a}} \sim N({\boldsymbol{0}}, G\sigma ^2_a)$ represents additive effects, ${\boldsymbol{g}} \sim N({\boldsymbol{0}}, \varSigma _g\sigma ^2_{g})$ captures non-additive effects, ${{\boldsymbol{g}}}{{\boldsymbol{e}}} \sim N({\boldsymbol{0}}, \text {diag}(e) \otimes I)$ represents genotype by environment interaction, and $\boldsymbol{\epsilon } \sim N({\boldsymbol{0}}, \text {diag}(e) \otimes I)$ represents residuals with heterogeneous variance across environments. The variance component for effects with non-diagonal covariance structures was estimated using the PEV methodology (Fernández-González et al. 2025).

Here, *G* is the VanRaden (2008) additive relationship matrix. We tested two different covariance structures for the $Z_{g} {\boldsymbol{g}}$ term: $\varSigma _g = I$, allowing the term $Z_{g} {\boldsymbol{g}}$ to capture all non-additive effects; and an epistatic relationship matrix(Henderson 1985; Gianola and de Los 2008), $\varSigma _g = G \circ G$, which restricts $Z_{g} {\boldsymbol{g}}$ to capture only epistatic effects. *I* is the identity matrix, and diag*(e)* refers to a diagonal covariance structure among environments. $X_e$, $X_{admix}$, $X_p$ $Z_a$, $Z_{g}$, and $Z_{ge}$ are the design matrices of their corresponding effects. The matrix $X_{admix}$ contains the admixture profile of each genotype, specifying the proportion of the genome corresponding to each subpopulation in this highly structured dataset. This term was included to better account for the potential impact of different linkage disequilibrium patterns and genetic backgrounds across subpopulations.

Environment and block effects were treated as fixed due to the limited number of levels, which would be insufficient for reliable variance estimation if treated as random (Gomes 2022). The variance of the fixed effect was calculated as the sample variance of the vector obtained by multiplying the design matrix by the vector of BLUEs. Variance components for the random effects were derived as described for the sunflower dataset, with similar considerations applied when splitting a variance component into smaller sources of variability.

The models using $\varSigma _g = I$ and $\varSigma _g = G \circ G$ had very similar AIC values but produced significantly different variance components for $\sigma ^2_{g}$: the former yielded very small values, while the latter resulted in $\sigma ^2_{g}$ being almost as large as $\sigma ^2_{a}$. Despite extremely similar broad-sense heritability in both cases, narrow sense heritability differed substantially. To resolve this discrepancy, we consulted the literature (Pfahler 1971; Tanvi and Jindal 2017; Jaiswal et al. 2022) to find a likely middle ground for narrow sense heritability. We then extracted the epistatic variance corresponding to that heritability value and used it for the subsequent simulations.

## Appendix 3: Simulation details

This simulation was based mainly on the “ESS simulation” from Fernández-González et al. (2025). Technow et al. (2012) and Melchinger and Frisch (2023) were also used as sources for sampling marker effects. The simulation relied on the empirical genome-wide markers for all the datasets. As the sunflower dataset contained only 224 fully genotyped hybrids, it was augmented with additional hybrids from the same breeding population that were not present in the field trials. This allowed us to obtain a population of 780 genotyped hybrids to use in the simulation.

### Simulation philosophy

An ideal simulation that mimics an empirical dataset should comply with two conditions: (i) have the same marker scores as the empirical dataset, (ii) have a realistic trait architecture. Unfortunately, to our knowledge, no current methodology is able to meet both conditions at the same time. The main problem is the Bulmer effect (Bulmer 1971), i.e., the negative LD induced between QTL in populations under selection. Standard simulations typically sample both the marker scores and the QTL effects in a random base population, and then simulate several generations of crossing and selection to generate a realistic trait architecture. However, this methodology has the drawback of generating a generic breeding population instead of the specific empirical one we are interested in. The alternative is starting with the empirical marker data of the desired population, which ensures that the desired population structure and LD patterns between marker positions is maintained. Then, some of the markers can be sampled to act as QTL and effects assigned to them. This, however, has the drawback that it produces a suboptimal trait architecture: there is no way to ensure the desired Bulmer effect is induced into the trait.

When a multi-generation breeding program is being simulated, it is critical to apply the standard simulation methodology that builds the marker scores *de novo*, as it is essential to have realistic Bulmer effect (otherwise genetic variance can suddenly drop by a massive amount once selection is introduced). However, in this work, we were focusing on single-generation field trials, and no recombination events were ever simulated. Under these circumstances, having a realistic Bulmer effect becomes less important. Firstly, genetic variance will always remain constant regardless of the Bulmer effect. Secondly, it could be argued that the negative LD between QTL is important for evaluating genomic prediction tools, as predictive models can be negatively impacted by multicollienarity. However, multicollinearity is equally present in both simulation strategies, simply in the standard approach it is predominantly negative due to the Bulmer effect, and in the alternative one both positive and negative correlations are equally possible. In both cases multicollinearity should be similarly detrimental for the model. Therefore, we believe that in the circumstances of the current work, preserving the population structure and LD patterns between marker scores from the empirical data is more important that obtaining a realistic Bulmer effect, as the former can have a large impact on genomic prediction accuracy while the latter probably does not.

As discussed above, we decided to use the empirical marker scores as the starting point for the simulation, sample some SNPs as QTL, and assign effects to them. Usually, the SNPs acting as QTL would be removed from the marker matrix used to optimize experimental designs and calibrate genomic prediction models. However, in the pipeline described in Fig. 1, this would have a massive computational cost. Under these circumstances, each simulation run would have a different genomic relationship matrix, thus requiring a specific experimental design optimization step. This would result in 300 different optimization runs per scenario, which would be unfeasible. Instead, we did not mask the QTL from the genomic prediction models or optimization steps. This allowed us to make the optimization step and the simulation completely independent: we could first find an optimal design and then build 10 independent simulations for it, massively reducing the number of times optimization had to be run. Of course, this has the drawback that the genomic prediction models now have access to the true QTL. This would be completely unacceptable for multi-generation simulations, in which it is critical to simulate the changes in LD induced by recombination between the QTL and the markers. However, in the single-generation scenario, we believe it is not problematic. In breeding populations genotyped with dense markers, it is extremely common for any marker to be very strongly correlated to at least one other marker. Under these circumstances, if one marker is acting as a QTL and is masked from the model, its effect is simply assigned to the correlated marker. If it is not masked from the model, its effect is divided between the actual QTL and the correlated marker. In any case we get the same accuracy for the model. As a result, we did not mask the SNPs acting as QTL, as we argue that there is no need to do so for comparing the accuracy of different genomic prediction strategies within a single generation. In a more mathematical notation, let $X_{QTL}$ be a matrix with genotypes in rows and QTL in columns, $X_m$ be a marker matrix with similar layout and $X_{all}$ be the column-wise concatenation of $X_{QTL}$ and $X_{m}$. For any vector of QTL effects $\boldsymbol{\alpha }$, the true additive values are $\boldsymbol{GVs} = X_{QTL}\boldsymbol{\alpha }$. From them, marker effects ($\boldsymbol{\beta _m}$) can be obtained as $\boldsymbol{\beta _m} = X_m'(X_mX_m')^{-1}\boldsymbol{GVs}$. Similarly, effects for all SNPs in $X_{all}$ can be obtained as $\boldsymbol{\beta _{all}} = X_{all}'(X_{all}X_{all}')^{-1}\boldsymbol{GVs}$. Genome-wide predictions from $X_m$ and $X_{all}$ are exactly equal ($X_{m}\boldsymbol{\beta _{m}} = X_{all}\boldsymbol{\beta _{all}} = \boldsymbol{GVs}$); the effects of the different loci get rearranged distinct ways when employing $X_m$ or $X_{all}$ but they result in the same predicted values. This holds as long as predictions are kept within the population of genotypes encompassed by $X_{all}$, but it no longer does if other individuals are predicted, as recombination events alter the correlations between loci. This showcases how, for genome-wide predictions within a single generation, it is not particularly important whether or not the true QTL are masked from the genomic prediction model. Therefore, due to the computational advantages of not masking the QTL, we decided not to do it.

Regarding the control of variance components, we employed the simulation methodology from Fernández-González et al. (2025), , which meticulously reflects the assumed genetic and non-genetic control of the phenotype. To our knowledge, it is also the only currently available methodology that incorporates the standard error of the variance. The latter refers to the fact that the variance components used as inputs for the simulation will be considered the true population variance, while each simulation run will simply be a sample from it, whose sample variance will be centered around the population variance but will not always be exactly the same as it. This allows for a very good exploration of different, likely combinations of variance components. Controlling this requires the use of a generalized equation for the standard error of the sample variance (SEV) that is capable of accounting for correlated samples. SEV can be computed as in Fernández-González et al. (2025) for any multivariate normal distribution ${\boldsymbol{X}} = \{X_1, X_2,..., X_n\} \sim N(\boldsymbol{\mu }, \varSigma )$:

9 $$\begin{aligned} \begin{array}{cc} SEM = \left( \frac{\bar{\sigma ^2}}{ESS_\mu }\right) ^{1/2} = \frac{(\boldsymbol{1^T}\varSigma {\boldsymbol{1}})^{1/2}}{n} \\ SEV = \frac{2^{1/2}\bar{\sigma ^2}}{(ESS_{\sigma ^2}-1)^{1/2}} = \frac{(\boldsymbol{1^T}\varSigma _2{\boldsymbol{1}})^{1/2}}{n-1} \\ \bar{\sigma ^2} = \frac{tr(\varSigma )}{n} \\ \varSigma _{2}[i,j] = 2(\varSigma _1[i,j])^2 + 4\varSigma _1[i,j] (\mu _i-\bar{\mu })(\mu _j-\bar{\mu }) \\ \varSigma _{1}[i,j] = \varSigma [i,j] + SEM^2 - \frac{\varSigma [i,1:n]{\boldsymbol{1}}}{n} -\frac{\varSigma [j,1:n]{\boldsymbol{1}}}{n} \\ ESS_\mu = \frac{tr(\varSigma )}{\boldsymbol{1^T}\varSigma {\boldsymbol{1}}} n \\ ESS_{\sigma ^2} = \frac{2(\bar{\sigma ^2})^2(n-1)^2}{\boldsymbol{1^T}\varSigma _2{\boldsymbol{1}}} + 1 \end{array} \end{aligned}$$

Where *SEM* refers to the standard error of the mean, $ESS_\mu$ is the effective sample size for the calculation of the sample mean, *SEV* is the standard error of the variance, and $ESS_{\sigma ^2}$ is the effective sample size for the calculation of the sample variance.

### Hybrid crops

The additive, additive by environment, and residual terms from Eq. 1 were simulated as described in Fernández-González et al. (2025). Raw marker effects ($\boldsymbol{\beta _a^*}$) were sampled from a gamma distribution as in Technow et al. (2012) for 3000 randomly sampled markers acting as quantitative trait loci (QTL). The additive variance value for any given simulation repetition ($s^2_a$) was sampled from a gamma distribution centered around the target additive variance ($\sigma ^2_a$, a parameter from Table 4) and with a standard deviation equal to $SEV_{a}$ (calculated from Eq. 9 with $\boldsymbol{\mu }$ equal to a vector of zero means, Σ equal to the *G* matrix, and $\bar{\sigma ^2} = \sigma ^2_a$):

$$\begin{aligned}s^2_a \sim \varGamma (\mu = \sigma ^2_a, \sigma ^2 = SEV_{a}^2)\end{aligned}$$

The marker effects were scaled in such a way that the variance of the additive values of the genotypes was equal to the sampled additive variance:

$$\begin{aligned} \boldsymbol{a^*} = M\boldsymbol{\beta _a^*} \end{aligned}$$

10 $$\begin{aligned} \boldsymbol{\beta _a} = \boldsymbol{\beta _a^*} \frac{s_a}{sd(\boldsymbol{a^*})} \end{aligned}$$

Where $\boldsymbol{a^*}$ is the vector of non-scaled additive values for all simulated genotypes. *M* is a numeric marker matrix counting the number of copies of the minor allele each genotype (row) has in each marker position (column). $\boldsymbol{\beta _a^*}$ is the vector of raw marker effects before scaling. $\boldsymbol{\beta _a}$ is the vector of scaled marker effects. This scaling ensures that $var(M\boldsymbol{\beta _a}) = s^2_a$.

The $g_i$ element from Eq. 1 corresponds to dominance values in hybrid crops. Here, we will describe how we simulated the vector of dominance values ${\boldsymbol{d}}$. First, dominance marker effects were sampled as described in Technow et al. (2012). For the $l^{th}$ QTL among the 3000 with additive effects, a dominance intensity value was sampled as follows $b^*_{d_i} \sim N(\mu = 1, \sigma = \sqrt{0.75})$. Using a mean for the normal distribution larger than zero ensures that most dominance will have a positive sign, i.e., there will be heterosis. The dominance effect for the $l^{th}$ marker ($\beta ^*_{d_l}$) can be computed from the dominance intensity values ($b^*_{d_l}$) and the additive marker effect ($\beta _{a_l}$) as follows: $\beta ^*_{d_l} = |\beta _{a_l}| b^*_{d_l}$, where |·| indicates that the absolute value is taken. Next, following Technow et al. (2012), 500 additional markers were sampled as QTL with dominance effects but without additive effects. Their dominance effects were sampled from $\beta ^*_{d_l} \sim N(\mu = 1, \sigma = \sqrt{0.75})$. The dominance effects for the 3500 total markers were merged into a single vector ($\boldsymbol{\beta ^*_{d}}$). Then, the dominance values for the hybrids can be computed as $\boldsymbol{d^*} = M_d \boldsymbol{\beta ^*_{d}}$, where $M_d$ is a marker matrix with hybrids in the rows and QTL in the columns that has a value of 1 for heterozygous positions and 0 for homozygous positions. $\boldsymbol{d^*}$ has an arbitrary variance, and it must be scaled to the target, which can be done as follows:

$$\begin{aligned} \boldsymbol{\beta _d} = \boldsymbol{\beta ^*_d} \frac{s_d}{sd(\boldsymbol{d^*})}; s^2_d \sim \varGamma (\mu = \sigma ^2_d, \sigma ^2 = SEV_{d}^2) \\ {\boldsymbol{d}} = M_d \boldsymbol{\beta _{d}} \end{aligned}$$

Where ${\boldsymbol{d}}$ is a vector of dominance values scaled to the desired variance, $\boldsymbol{\beta _d}$ are the scaled dominance marker effects, $sd(\boldsymbol{d^*})$ is the standard deviation of the vector $\boldsymbol{d^*}$, $\varGamma (\mu = \sigma ^2_a, \sigma _d^2 = SEV_{d}^2)$ is a gamma distribution from which $s^2_d$ is sampled, $\sigma ^2_d$ is the target dominance variance for the population (user-defined parameter taken from Table 4), and $SEV_{d}$ is the standard error of the variance calculated from Eq. 9 with Σ equal to a Vitezica et al. (2013) dominance relationship matrix.

Next, we will describe the simulation for the environmental effects and their interaction with genotype (terms $e_j$, $ae_{ij}$, and $ge_{ij}$ in Eq. 1). For the $ae_{ij}$ term, we need a matrix *AxE* that contains all interaction effects between all genotypes and all environments. Similarly, for $ge_{ij}$, we need a *DxE* term containing the interaction between dominance and environments. The main environmental effect $e_j$ can be obtained from the average interaction effects across all genotypes for environment *j*. To obtain these values, we first had to assume a variance-covariance matrix between environments (*K*). We sampled the diagonal elements of *K* from $N(\mu = 1.25, \sigma = 0.25)$. The correlations between environments were sampled from $N(\mu = 0.2, \sigma = 0.20)$ and bounded between -1 and 1. The resulting *K* matrix was then approximated to its nearest positive definite matrix. Next, 100 environmental covariates were sampled for each environment from $N({\boldsymbol{0}}, K)$, and the results were stored in a matrix ($E_{cov}$) with the environmental covariates in the rows and the environments in the columns.

Next, we created a matrix of the intensity of interaction between additive effects and environmental covariates ($B_{ae}$). $B_{ae}$ had one row per QTL and one column per environmental covariate, and we sampled its elements from a standard normal distribution. We also had to define the matrix $M_{\beta _a}$, which contains the additive effects for each genotype (rows) and QTL (columns):

$$\begin{aligned} M_{\beta _a} = M \begin{pmatrix} \beta _{a_1} & \quad 0 & \quad \dots & \quad 0 \\ 0 & \quad \beta _{a_2} & \quad & \quad 0 \\ \vdots & \quad & \quad \ddots & \quad \vdots \\ 0 & \quad 0 & \quad \dots & \quad \beta _{a_p} \end{pmatrix} \end{aligned}$$

The non-scaled additive by environment interaction matrix $AxE^*$ was obtained by multiplying each marker effect in $M_{\beta _a}$ by each environmental covariate in $E_{cov}$ and their interaction intensity in $B_{ae}$, and adding up the corresponding values for each genotype-environment combination. In matrix notation:

11 $$\begin{aligned} AxE^* = M_{\beta _a} B_{ae} E_{cov} \end{aligned}$$

Next, the $AxE^*$ was double-centered to remove any main effects and leave only the interaction terms. Finally, the double-centered matrix ($AxE^*_c$) was scaled to the target variance as:

$$\begin{aligned} &  AxE = AxE_c^* \frac{s_{ae}}{sd(AxE_c^*)}; s^2_{ae} \\ &  \quad \sim \varGamma (\mu = \sigma ^2_{ae}, \sigma ^2 = SEV_{ae}^2) \end{aligned}$$

The $ae_{ij}$ values from Eq. 1 are instances of the *AxE* matrix. $sd(AxE_c^*)$ refers to computing the sample standard deviation for all elements within the $AxE_c^*$ matrix. $SEV_{ae}$ was computed from Eq. 9 with Σ equal to the Kronecker product of *K* and *G*. $\sigma ^2_{ae}$ is the additive by environment variance from Table 4. The dominance by environment interaction effects were calculated similarly, replacing $M_{\beta _a}$ with a matrix $M_{\beta _d}$ containing genotypes in rows and QTL in columns, with elements that represent the dominance effects for each QTL in each individual. The $B_{ae}$ matrix was also replaced by a similar matrix $B_{de}$ sampled from a standard normal distribution and with the required dimensions:

12 $$\begin{aligned} DxE^* = M_{\beta _d} B_{de} E_{cov} \end{aligned}$$

$$\begin{aligned} &  DxE = DxE_c^* \frac{s^2_{de}}{sd(DxE_c^*)}; s^2_{de} \sim \varGamma (\mu \\ &  \quad = \sigma ^2_{de}, \sigma ^2 = SEV_{de}^2) \end{aligned}$$

The $ge_{ij}$ values from Eq. 1 are instances of the *DxE* matrix. $DxE_c^*$ is the double-centered $DxE^*$ matrix. $SEV_{de}$ was computed from Eq. 9 with Σ equal to the kronecker product between *K* and the dominance relationship matrix. $\sigma ^2_{de}$ was the dominance of environmental variance from Table 4.

The non-scaled environmental main effects $\boldsymbol{e^*}$ were calculated as the sum of the column means of $AxE^*$ and $DxE^*$, scaled to $s^2_{ae}$ and $s^2_{de}$ variance, respectively. Then, the scaled environmental effects (${\boldsymbol{e}}$) were calculated as:

13 $$\begin{aligned} {\boldsymbol{e}} = \boldsymbol{e^*} \frac{s^2_{e}}{sd(\boldsymbol{e^*})}; s^2_{e} \sim \varGamma (\mu = \sigma ^2_{e}, \sigma ^2 = SEV_{e}^2) \end{aligned}$$

Where $\sigma ^2_{e}$ was the environmental variance from Table 4, and $SEV_{e}$ was computed from Eq. 9 with Σ=K.

Plot effects can be computed similarly to the environmental effects. Just as 100 environmental covariates were sampled for each environment, 100 environmental covariates can be sampled for each plot. This allows for the computation of the main spatial effects for each plot, additive by spatial and dominance by spatial interactions, using the same equations as those used for the main environmental effects and their interactions with the genotype. To sample the spatial effects within each environment *j*, we assumed that the plots followed a covariance matrix equal to a two-dimensional autocorrelation matrix of order one:

14 $$\begin{aligned} &  AR1xAR1_j = \begin{pmatrix} 1 & \quad \rho _{rows,j} & \quad \rho ^2_{rows,j} & \quad ... & \quad \rho ^{r-1}_{rows,j} \\ \rho _{rows,j} & \quad 1 & \quad \rho _{rows,j} & \quad ... & \quad \rho ^{r-2}_{rows,j} \\ \rho ^2_{rows,j} & \quad \rho _{rows,j} & \quad 1 & \quad ... & \quad \rho ^{r-3}_{rows,j} \\ \vdots & \vdots & \vdots & \ddots & \vdots \\ \rho ^{r-1}_{rows,j} & \quad \rho ^{r-2}_{rows,j} & \quad \rho ^{r-3}_{rows,j} & \quad ... & \quad 1 \end{pmatrix} \nonumber \\ &  \quad \otimes \begin{pmatrix} 1 & \rho _{cols,j} & \quad \rho ^2_{cols,j} & \quad ... & \quad \rho ^{c-1}_{cols,j} \\ \rho _{cols,j} & 1 & \quad \rho _{cols,j} & \quad ... & \quad \rho ^{c-2}_{cols,j} \\ \rho ^2_{cols,j} & \quad \rho _{cols,j} & \quad 1 & \quad ... & \quad \rho ^{c-3}_{cols,j} \\ \vdots & \vdots & \vdots & \ddots & \vdots \\ \rho ^{c-1}_{cols,j} & \quad \rho ^{c-2}_{cols,j} & \quad \rho ^{c-3}_{cols,j} & \quad ... & \quad 1 \end{pmatrix} \end{aligned}$$

Where $AR1xAR1_j$ is the correlation matrix across plots within the environment, *j*, $\rho _{rows,j}$, and $\rho _{cols,j}$ are the autocorrelation parameters, and *r* and *c* are the number of rows and number of columns, respectively. The values for $\rho _{rows,j}$ and $\rho _{cols,j}$ were sampled in each simulation iteration from:

$$\begin{aligned} \begin{pmatrix} \rho _{rows,j} \\ \rho _{cols,j} \end{pmatrix} \sim N \left( \boldsymbol{\mu } = \begin{pmatrix} 0.85 \\ 0.85 \end{pmatrix}, \varSigma = \begin{pmatrix} 0.0025 & 0.002 \\ 0.002 & 0.0025 \end{pmatrix} \right) \end{aligned}$$

A constraint was added that $\rho _{rows,j}$ and $\rho _{cols,j}$ had to be positive and smaller than 0.95. Please note that the chosen variance values of 0.0025 seem very small, but they result in a standard deviation of 0.05. This means that roughly 95% of the probability density for the autocorrelation values is within the interval (0.75,0.95) before applying any constraints.

We used this framework to simulate spatial effects and their interaction with the genotype ($p_k$, $ap_{ijk}$ and $gp_{ijk}$ in Eq. 1). The environmental covariates for the plots in environment *j* ($P_{cov,j}$) were sampled and used in a manner similar to the $E_{cov}$ matrix in Eqs. 11 and 12:

$$\begin{aligned}P_{cov,j} \sim N({\boldsymbol{0}}, AR1xAR1_j)\end{aligned}$$

15 $$\begin{aligned} AxP_j^* = M_{\beta _a} B_{ae} P_{cov,j} \end{aligned}$$

Where $AxP_j^*$ is the additive by spatial interaction in the $j^{th}$ field. Next, the $AxP_j^*$ was double-centered to remove any main effects and leave only the interaction terms. Finally, the double-centered matrix ($AxP_{c_j}^*$) was scaled to the target variance as:

$$\begin{aligned} AxP_j {=} AxP_{c_j}^* \frac{s_{ap}}{sd(AxP_{c_j}^*)}; s^2_{ap} \sim \varGamma (\mu {=} \sigma ^2_{ap}, \sigma ^2 {=} SEV_{ap}^2) \end{aligned}$$

The $ap_{ijk}$ values from Eq. 1 are instances of the $AxP_j$ matrix. $SEV_{ap}$ was computed from Eq. 9 with Σ equal to the kronecker product between $AR1xAR1_j$ and *G*. $\sigma ^2_{ap}$ is the additive based on spatial variance from Table 4. The dominance of spatial interaction effects was calculated similarly, replacing $M_{\beta _a}$ with a matrix $M_{\beta _d}$ and $B_{ae}$ with $B_{de}$:

16 $$\begin{aligned} DxP_j^* = M_{\beta _d} B_{de} P_{cov,j} \end{aligned}$$

$$\begin{aligned} DxP_j {=} DxP_{c_j}^* \frac{s_{dp}}{sd(DxP_{c_j}^*)}; s^2_{dp} \sim \varGamma (\mu {=} \sigma ^2_{dp}, \sigma ^2 {=} SEV_{dp}^2) \end{aligned}$$

The $gp_{ijk}$ values from Eq. 1 are instances of the $DxP_j$ matrix. $DxP_{c_j}^*$ is the double-centered $DxP_j^*$ matrix. $SEV_{dp}$ was computed from Eq. 9 with Σ equal to the kronecker product between $AR1xAR1_j$ and the dominance relationship matrix. $\sigma ^2_{dp}$ was the dominance of spatial variance from Table 4.

The non-scaled spatial main effects within environment *j* ($\boldsymbol{p_j^*}$) were calculated as the sum of the column means of $AxP_j^*$ and $DxP_j^*$, scaled to $s^2_{ap}$ and $s^2_{dp}$ variance, respectively. Then, the scaled environmental effects ($\boldsymbol{p_j}$) were calculated as:

17 $$\begin{aligned} \boldsymbol{p_j} = \boldsymbol{p_j^*} \frac{s^2_{p}}{sd(\boldsymbol{p_j^*})}; s^2_{p} \sim \varGamma (\mu = \sigma ^2_{p}, \sigma ^2 = SEV_{p}^2) \end{aligned}$$

Finally, residuals $\boldsymbol{\epsilon }$ were randomly sampled and added to each plot within each environment:

$$\begin{aligned} \boldsymbol{\epsilon } \sim N({\boldsymbol{0}}, I\sigma ^2_\epsilon ) \end{aligned}$$

Where *I* is the identity matrix of the necessary dimensions.

### Self-pollinated crop

For oats, the simulation was similar to that for the hybrid crops, but dominance was replaced by epistasis. Additionally, we simulated the fact that the marker effects can vary in different subpopulations due to differing linkage disequilibrium patterns with the actual causal QTL. To that end, we used the admixture matrix ($X_{admix}$), which contains genotypes in the rows and subpopulations in the columns, and its values represent the proportion of each genotype that belongs to each subpopulation. An admixture effect for each subpopulation was sampled from a standard normal distribution, and it was stored in the vector $\boldsymbol{\beta ^*_{admix}}$. The admixture values for each genotype would be: $\boldsymbol{Admix^*} = X_{admix}\boldsymbol{\beta ^*_{admix}}$. These values are scaled to the desired target variance as:

$$\begin{aligned} \boldsymbol{Admix}= &  \boldsymbol{Admix^*} \frac{s_{admix}}{sd(\boldsymbol{Admix^*})}; \\ &  s^2_{admix} \sim \varGamma (\mu = \sigma ^2_{admix}, \sigma ^2 = SEV_{Admix}) \end{aligned}$$

Where $\boldsymbol{Admix}$ is a vector of admixture effects scaled to the desired variance. $sd(\boldsymbol{Admix^*})$ is the standard deviation of the vector $\boldsymbol{Admix^*}$. $\sigma ^2_{admix}$ is the target admixture variance for the population (user-defined parameter taken from Table 4). The covariance matrix for $SEV_{Admix}$ computation was calculated from Eq. 9 with $\varSigma {=} \frac{Z_{admix}Z_{admix}'}{mean(diag(Z_{admix}Z_{admix}'))} + 10^{-6}I$, where $Z_{admix}$ is the result of double-centering the $X_{admix}$ matrix, and *I* is the identity matrix of the required dimensions.

Next, let *M* be a marker matrix containing 3000 randomly sampled markers (the markers chosen to have non-zero additive effects). It is known that additive genotypic values can be projected into marker effects (Henderson 1977) using the matrix $M'(MM')^{-1}$. Similarly, we can project the admixture values into marker effects as follows:

$$\begin{aligned} \boldsymbol{\beta _{admix}} = M'(MM')^{-1}\boldsymbol{Admix} \end{aligned}$$

Let $\boldsymbol{\beta }$ be the vector of additive marker effects obtained from Eq. 10. Then, the actual marker effects accounting for the distorting effect of population structure would be $\boldsymbol{\beta } = \boldsymbol{\beta _a} + \boldsymbol{\beta _{admix}}$. Subsequently, $\boldsymbol{\beta }$ was used instead of $\boldsymbol{\beta _a}$ when needed for the computation of the interaction terms.

Epistatic effects were simulated for 1000 random pairs of QTL. For each QTL pair *m*, comprised of the QTL $m_1$ and $m_2$, a value for the intensity of their epistatic interaction ($\beta aa^*_{m}$) was sampled from a standard normal distribution. Then, the epistatic effect between QTL $m_1$ and $m_2$ for individual *i* ($aa^*_{i,m}$) is:

$$\begin{aligned} aa^*_{i,m} = (M[i,m_1]\beta _{m_1}) (M[i,m_2]\beta _{m_2}) \beta aa^*_{m} \end{aligned}$$

Where $M[i,m_1]$ indicates that the marker score for individual *i* and QTL $m_1$ is extracted from the *M* matrix. $\beta _{m_1}$ and $\beta _{m_2}$ are the additive marker effects of the QTL $m_1$ and $m_2$. The overall epistasis value for a genotype *i* is simply the sum of the epistasis effects of all its marker pairs, i.e. $aa^*_{i} = \sum _{m=1}^{1000} aa^*_{i,m}$. These epistasis values are scaled to the desired target variance as follows:

$$\begin{aligned} \boldsymbol{\beta aa}= &  \boldsymbol{\beta aa^*}\frac{s_{aa}}{sd(\boldsymbol{aa^*})}; s_{aa} \sim \varGamma (\mu = \sigma ^2_{aa}, \sigma ^2 = SEV_{aa}^2) \\ aa_{i,m}= &  (M[i,m_1]\beta _{m_1}) (M[i,m_2]\beta _{m_2}) \beta aa_{m} \\ aa_i= &  \sum _{m=1}^{1000} aa_{i,m} \end{aligned}$$

Where $\boldsymbol{aa^*}$ is the vector of non-scaled epistatic values across all genotypes, $\boldsymbol{\beta aa^*}$ is the vector of non-scaled values for the intensity of epistatic interactions across all QTL pairs, $\boldsymbol{\beta aa}$ is its corresponding scaled vector, and $aa_i$ is the scaled epistatic value of genotype *i*, where $sd(aa_1, aa_2,..., aa_n) = s_{aa}$, i.e., we have forced them to have the target variance. $SEV_{aa}$ was computed from Eq. 9 with $\varSigma = G \circ G$. $\sigma ^2_{aa}$ is the epistatic variance extracted from Table 4.

Epistatic effects modulated by the environment were encoded in a matrix $M_{aa}\in {\mathbb {R}}^{n\times M}$, with genotypes in rows and all QTL pairs in columns; its entries are $M_{aa}[i,m]=aa_{i,m}$, the epistatic effect of the marker pair *m* in individual *i*. We sampled an interaction–loading matrix $B_{aae}\in {\mathbb {R}}^{M\times q}$ from a standard normal distribution to capture the intensity of interactions between epistatic marker pairs and the *q* environmental covariates. Then:

$$\begin{aligned} AAxE^* = M_{aa} B_{aae} E_{cov} \end{aligned}$$

$$\begin{aligned} &  AAxE = AAxE_c^* \frac{s_{aae}}{sd(AAxE_c^*)}; s_{aae} \\ &  \quad \sim \varGamma (\mu = \sigma ^2_{AAxE}, \sigma ^2 = SEV_{aa}^2) \end{aligned}$$

Where *AAxE* is the matrix of epistasis by environment interaction of each genotype with each environment, and $AAxE_c^*$ is the double-centered $AAxE^*$ matrix. $SEV_{aa}$ was computed from Eq. 9 with Σ equal to the kronecker product between *K* and the G∘G relationship matrix. $\sigma ^2_{AAxE}$ is the epistasis by environment variance from Table 4.

All other terms in the simulation for the oat dataset were computed similarly to those for hybrid crops, replacing dominance with epistasis when needed.

## Appendix 4: Tuning of the autocorrelation parameter

For the optimization of the spatial distribution of the genotypes in the field, we need two inputs: the relatedness between the genotypes and the relatedness between the plots in the field, i.e. the matrices *G* and *R* in Eq. 2 respectively. *G* is simply the genomic relationship matrix, but *R* is often not known. Instead, we can assume that *R* is an autoregressive correlation matrix computed as in Eq. 14. However, this requires tuning the values of the autocorrelation parameters $\rho _{rows}$ and $\rho _{cols}$. These parameters can be interpreted in two ways: i) they can be thought of as the actual autocorrelation between plots present in the field, or ii) they can be interpreted as parameters controlling how global or local the scope of the optimization is.

The former view of $\rho _{rows}$ and $\rho _{cols}$ would require performing expensive trials in the field to determine their value, which is often not possible (unless extensive historical data are readily available). Conversely, the latter interpretation for $\rho _{rows}$ and $\rho _{cols}$ simply focuses on obtaining values that are suitable for the optimization process, regardless of the actual spatial trends in the field. This allows for very easy tuning of the parameters. Very high autocorrelation values result in plots that are very distant in the field, having non-negligible correlations between them. As a result, during the optimization, the algorithm has to take into account the influence that very distant plots have on one another; i.e., the optimization is very global. In contrast, for very low autocorrelation values, only very close plots will exhibit non-negligible correlations. Therefore, the optimization algorithm will consider that, when optimizing which genotype should be placed in a plot, only the genotypes in the immediately neighboring plots need to be considered. Generally, a balance between these two extremes is desired to avoid having an optimization that is either too broad or has a scope that is too narrow. In preliminary testing, we found that this approach allowed for good performance in optimization, even if the selected values for $\rho _{rows}$ and $\rho _{cols}$ did not match the actual autocorrelation present in the simulated fields. Actually, the optimal values for $\rho _{rows}$ and $\rho _{cols}$ depended only on field size. In large fields, there is a lot of space between very distant plots, allowing the correlation between them to decay to very small values, even with relatively high values for $\rho _{rows}$ and $\rho _{cols}$. However, with the same autocorrelation values, even the most distant plots in a small field can be substantially correlated due to the lower distances involved. As such, the same values will result in relatively local optimization in the large field, but in very broad and global optimization in the small field. This is illustrated in Fig. 5. To solve this problem, we determined that the $\rho _{rows}$ and $\rho _{cols}$ should be tuned in such a way that the most distant plots (i.e., those in opposite corners of the field) had a correlation of 0.001, regardless of field size. We obtained this value in preliminary testing, and it allowed for consistent optimization performance.

It is important to highlight that the autocorrelation values obtained in this way will not match the actual autocorrelation present in the field; they are simply parameters used to control how local or global the optimization is. To ensure a fair assessment, autocorrelation was estimated independently for simulation and optimization, yielding markedly different values. Accordingly, the reported optimization performance already incorporates uncertainty in the field’s true autocorrelation.

Finally, we will explain how the values of $\rho _{rows}$ and $\rho _{cols}$ that cause the minimum correlation to be 0.001 were obtained. From Eq. 14, it becomes clear that in a rectangular field, the minimum correlation between two plots will be $\rho _{rows}^{r-1}\rho _{cols}^{c-1}$, where *r* is the number of rows and *c* is the number of columns. Therefore:

$$\begin{aligned} 0.001 = \rho _{rows}^{r-1}\rho _{cols}^{c-1} \end{aligned}$$

$$\begin{aligned} \rho _{rows} = \rho _{cols}^k \end{aligned}$$

Where $k = \frac{d_r}{d_c}$; $d_r$ is the distance between the centers of two adjacent plots in the same column and different rows, and $d_c$ is the distance between the centers of two adjacent plots in the same row and different columns. Rearranging and taking logarithms, we can solve the system of equations:

$$\begin{aligned} \rho _{cols} = e^{\frac{log(0.001)}{k(r-1)+(c-1)}} \end{aligned}$$

$$\begin{aligned} \rho _{rows} = e^{\frac{log(0.001)}{(r-1)+(c-1)/k}} \end{aligned}$$

In this work, we assumed perfectly square plots, which result in $k=1; \rho _{rows} = \rho _{cols} = \rho$. However, if the plots are rectangular rather than square, the ratio may differ from one. The longer the plots in a given direction, the longer the distance needed to accommodate a given number of plots in that direction, and the lower the autocorrelation value.

## Appendix 5: CDmean computational efficiency

Optimization of the distribution of genotypes in the field using genomic information typically relies on model-based, parametric criteria such as A-optimality (Cullis et al. 2006). These criteria rely on the calculation of the PEV matrix, which is an important bottleneck in terms of computational efficiency. It is important to note that during optimization, a heuristic has to compute the optimization criteria many thousands of times. Therefore, the PEV matrix must be computed repeatedly, making it imperative to minimize its computational cost. Feoktistov et al. (2017) implemented a differential-evolution algorithm with good convergence but relied on slow PEV evaluations, limiting scalability. Butler et al. (2022) addressed this by updating the PEV matrix at each iteration rather than recomputing it *de novo*, following Martin and Eccleston (1992). This strategy is efficient only when successive updates are small. This limits the choice of heuristics to simple algorithms, such as tabu search (Butler et al. 2022). However, we believe that this simple heuristic may have trouble exploring the search space for relatively large plots, where the number of possible solutions is astronomical. Ideally, it would be desirable to use a more complex heuristic, such as in Feoktistov et al. (2017), while minimizing the cost of computing PEV, as in Butler et al. (2022). To that end, we chose the TrainSel heuristic (Akdemir et al. 2021), which is a computationally efficient C++ implementation of a genetic algorithm combined with simulated annealing. It has the further advantage of accepting any user-defined optimization criteria, which allows us to easily adjust the PEV computation to maximize computational efficiency. To explain our approach, we will first describe the typical method of computing the PEV matrix, and then we will show the improvements we have implemented. We will assume that the underlying model in both cases is ${\boldsymbol{y}} = X\mu + Z{\boldsymbol{a}} + \boldsymbol{\epsilon }$, as explained in the main text, Eq. 2, which is the most commonly assumed model for optimization.

### Typical PEV computation

PEV is computed from the coefficient matrix of the Henderson equations (Henderson 1975) as explained in Eq. 2 of the main text. There are several matrix multiplications and inversions with cubic time complexity that slow down this process:

- Matrix multiplications involving the design matrix of the fixed effects (*X*). Its computational cost is negligible because, in the assumed model, *X* is a vector of ones, thus having very small dimensions.
- Matrix multiplications involving the design matrix of the random effects (*Z*). While this matrix is large, it is also very sparse, making the computational cost negligible.
- Inversion of the covariance matrices *R* and *G*. While this is computationally costly, it can be done a single time at the beginning and provided as input to the heuristic. Then, the heuristic will calculate PEV thousands of times without having to invert *R* or *G* again, making their computational costs negligible.
- Inversion of the coefficient matrix. This is the main bottleneck for computational speed.

Thus, it is critical to maximize the computational efficiency of the inversion of the coefficient matrix. From Eq. 2, it becomes clear that we do not need the full inverse; we only need its bottom-left block $C_{22} = PEV$. This can be calculated without computing the full inverse as follows (Henderson et al. 1984):

18 $$\begin{aligned} \begin{array}{c} C_{22} = (Z'MZ + \lambda G^{-1})^{-1} \sigma ^2_\epsilon \\ M = V^{-1} - V^{-1}X(X'V^{-1}X)^{-1}X'V^{-1} \end{array} \end{aligned}$$

Where $$\lambda = \frac{\sigma ^2_\epsilon }{\sigma ^2_a}$$, *M* is a projection matrix that allows for including the influence of the fixed effects during the matrix inversion, and *V* is the phenotypic variance under the assumed model, i.e., $$V = ZG\sigma ^2_a Z' + R\sigma ^2_\epsilon $$. This is often simplified to V=I if R=I (Henderson et al. 1984). When V=I-as is often the case in training-set optimization (Rincent et al. 2012)-the cost of computing *M* is negligible. By contrast, optimizing the spatial arrangement of genotypes in the field typically requires modeling *R* as an $$\textrm{AR1}\times \textrm{AR1}$$ covariance (Eq. 14), which, in turn, necessitates computing$V=ZGZ^{\prime }+R,$, substantially increasing the computational burden. Here, the inverse of *V* will have different values depending on the *Z* matrix; i.e., it will vary depending on the spatial distribution of the genotypes in the field. As a result, we cannot pre-calculate the $$V^{-1}$$ matrix at the beginning, and the heuristic will have to compute it thousands of times, dramatically increasing the computational cost.

In general, the computational cost of computing PEV this way is determined by its two bottlenecks, inverting *V* and inverting $(Z'MZ + \lambda G^{-1})$. The former has dimensions p×p, and the latter has dimensions n×n, where *p* and *n* are the numbers of plots and genotypes, respectively. Given that, due to replication, *p* is almost always larger than *n*, and that matrix inversion has cubic time complexity, $p^3>> n^3$, the inversion of *V* being the main limitation for the optimization process. In summary, given that the number of floating point operations (flops) needed to invert a matrix is the cube of its dimensions, the flops required for computing PEV from Eq. 18 is approximately $p^3 + n^3$.

### Improved PEV computation

In this work, we propose an alternative method for computing PEV, avoiding the need for the costly inversion of the *V* matrix. To that end, we simply invert the full coefficient matrix and subset $C_{22}$ from it, as shown in Eq. 2. The equation for the full coefficient matrix is: $$CoeffMat = \begin{pmatrix} X'(R\sigma ^2_\epsilon )^{-1}X & X'(R\sigma ^2_\epsilon )^{-1}Z Z'(R\sigma ^2_\epsilon )^{-1}X & Z'(R\sigma ^2_\epsilon )^{-1}Z \\ + (G\sigma ^2_a)^{-1} \end{pmatrix}$$. *CoeffMat* seems very large, but it actually has dimensions (n+1)×(n+1) due to the fact that *X* is only a vector of ones. This is only marginally larger than the n×n matrix needed in Eq. 18, while it does not require the expensive inversion of the *V* matrix. Consequently, the floating-point operations required to compute the PEV in this manner are only $(n+1)^3$, which is much smaller than the $p^3 + n^3$ cost of the classical approach.

However, the $(n+1)^3$ cost is still high and can be further reduced in some cases. The typical optimization algorithm for the spatial distribution of the genotypes is A-optimality (Cullis et al. 2006; Butler et al. 2022), which relies on minimizing *A*:

$$\begin{aligned} A = \frac{2}{n-1}\left( tr(PEV)-\frac{1}{n} \boldsymbol{1'}PEV{\boldsymbol{1}}\right) \end{aligned}$$

Where *tr*() stands for the trace of a matrix. Computing *A* requires the full *PEV* matrix, precluding any further computational gains. This is the reason why, instead of A-optimality, we chose the maximization of CDmean, which only requires the main diagonal of the PEV matrix:

$$\begin{aligned} CDmean = \boldsymbol{w'}\frac{diag(G\sigma ^2_a) - diag(PEV)}{diag(G\sigma ^2_a)} \end{aligned}$$

This opens up the possibility of computing only the main diagonal of the inverse of the coefficient matrix instead of the full inverse, which can be achieved through a Cholesky decomposition:

$$\begin{aligned} CoeffMat = LL'; CoeffMat^{-1} = L^{-1}(L')^{-1} \end{aligned}$$

Where *L* is a lower triangular matrix computed through the Cholesky decomposition. The $i^{th}$ diagonal element of $CoeffMat^{-1}$ can be computed as follows:

$$\begin{aligned} CoeffMat^{-1}[i,i] = \sum _{j=1}^{(n+1)} L[i,j]^2 \end{aligned}$$

Where [*i*, *j*] indicates that we have taken the element in the $i^{th}$ row and $j^{th}$ column of a matrix. This method is very computationally efficient. A typical full inversion of the (n+1)×(n+1) coefficient matrix requires $(n+1)^3$ flops. Conversely, the Cholesky decomposition only requires $(n+1)^3/3$ operations; the inversion of *L* by forward substitution requires $(n+1)^2$ flops, and the computation of the diagonal elements of PEV as $PEV[i,i] = \sum _{j=1}^{(n+1)} L[i,j]^2$ requires $2(n+1)^2$ flops. Therefore, the full computational cost is $(n+1)^3/3 + (n+1)^2 + 2(n+1)^2 \approx (n+1)^3/3$. This means that finding only the main diagonal of PEV (flops = $(n+1)^3/3$) can be three times faster than finding the entire PEV matrix (flops = $(n+1)^3$).

In summary, computing the optimization criterion using the classical method of calculating PEV (Eq. 18) costs approximately $n^3+p^3$ flops, which is much larger than the cost of the proposed CDmean criterion (Eq. 2; $(n+1)^3/3$ flops).

### Targeted optimization acceleration

In this work, we performed targeted optimization, i.e., we considered the genotypic information of the TS individuals when optimizing the spatial allocation of the TRS genotypes. This has massive computational implications. If there are *nTS* individuals in the TS and *nTRS* individuals in the TRS, the coefficient matrix in Eq. 2 becomes (nTS+nTRS+1)×(nTS+nTRS+1). While *nTRS* is limited to being smaller than *p*, *nTS* can be extremely large if we desire to predict the performance of numerous genotypes. This can make the coefficient matrix too large for the optimization. Therefore, instead of including all *nTS* individuals for the optimization, we used the Avg_GRM_self algorithm (which is very fast) to select 100 representative individuals from the *TS* and included only them for the optimization, resulting in the coefficient matrix (nTRS+101)×(nTRS+101), which is manageable. However, this reduction in the number of *TS* individuals decreases their weight in the overall value of CDmean, which is undesirable. To compensate for this, we used the vector ${\boldsymbol{w}}$ from Eq. 2. ${\boldsymbol{w}}$ allows control of the relative weight of the TRS and TS in optimization. We set the weight $\frac{1}{nTRS+nTS}$ for each TRS genotype and $\frac{nTS/100}{nTRS+nTS}$ for the TS individuals. This compensates for the reduction in weight of the TS due to including only 100 of them in the optimization.

Please note that the ${\boldsymbol{w}}$ allows for more possibilities, such as giving zero weight to TRS individuals and focusing on maximizing the reliability of the predictions of the TS. However, in preliminary testing, we found that this strategy was suboptimal. Any user-defined weights can be used to give more relevance to important genotypes, although we have not tested that option, and its impact on accuracy remains unknown.

## References

[CR1] Ahmed M, Morad KA, Attia M, Ghareeb ZE (2020) Study the seed and oil yield stability of sunflower hybrids across environments. Asian J Adv Res Rep 13(2):28–42

[CR2] Akdemir D, Isidro-Sánchez J (2019) Design of training populations for selective phenotyping in genomic prediction. Sci Rep 9(1):1446 30723226 10.1038/s41598-018-38081-6 PMC6363789

[CR3] Akdemir D, Rio S, Sánchez IY et al (2021) TrainSel: an R package for selection of training populations. Front Genet 12:607 10.3389/fgene.2021.655287 PMC8138169 34025720

[CR4] Alemu A, Åstrand J, Montesinos-Lopez OA, Sánchez IY, Fernandez-Gonzalez J, Tadesse J, Vetukuri W, Carlsson RR, Ceplitis AS, Crossa AJ (2024) Genomic selection in plant breeding: key factors shaping two decades of progress. Mol Plant 17:552–578 38475993 10.1016/j.molp.2024.03.007

[CR5] Bulmer M (1971) The effect of selection on genetic variability. Am Nat 105(943):201–211

[CR6] Butler D, Smith A, Cullis B (2022) On model based design of comparative experiments. University of Wollongong, Wollongong, National Institute for Applied Statistics Research Australia

[CR7] Canales FJ, Montilla-Bascón G, Bekele WA, Howarth C, Langdon T, Rispail N, Tinker N, Prats E (2021) Data set from: population genomics of mediterranean oat (a. sativa) reveals high genetic diversity and three loci for heading date.(Dryad Dataset) 10.1007/s00122-021-03805-2 PMC8263550 33770189

[CR8] Cappa EP, Muñoz F, Sanchez L (2019) Performance of alternative spatial models in empirical douglas-fir and simulated datasets. Ann For Sci 76(2):53

[CR9] Carvalho IR, Nardino M, Demari GH, de Pelegrin AJ, Ferrari M, Szareski VJ, de Oliveira VF, Barbosa MH, de Souza VQ, de Oliveira AC et al (2017) Components of variance and inter-relation of important traits for maize (‘Zea mays’) breeding. Aust J Crop Sci 11(8):982–988

[CR10] Casler MD (2015) Fundamentals of experimental design: guidelines for designing successful experiments. Agron J 107(2):692–705

[CR11] Clark SA, Hickey JM, Daetwyler HD, van der Werf JH (2012) The importance of information on relatives for the prediction of genomic breeding values and the implications for the makeup of reference data sets in livestock breeding schemes. Genet Sel Evol 44(1):4 22321529 10.1186/1297-9686-44-4 PMC3299588

[CR12] Coutiño-Estrada B, Vidal-Martínez VA (2006) Variance components of corn hybrids evaluated in the USA corn belt. Agrociencia 40(1):89–98

[CR13] Covarrubias-Pazaran G (2016) Genome assisted prediction of quantitative traits using the r package sommer. PLoS ONE 11:1–15 10.1371/journal.pone.0156744 PMC4894563 27271781

[CR14] Cullis BR, Smith AB, Coombes NE (2006) On the design of early generation variety trials with correlated data. J Agric Biol Environ Stat 11(4):381–393

[CR15] Damesa TM, Hartung J, Gowda M, Beyene Y, Das B, Semagn K, Piepho H-P (2019) Comparison of weighted and unweighted stage-wise analysis for genome-wide association studies and genomic selection. Crop Sci 59(6):2572–2584

[CR16] Damesa TM, Möhring J, Worku M, Piepho H-P (2017) One step at a time: stage-wise analysis of a series of experiments. Agron J 109(3):845–857

[CR17] Eilers PH, Marx BD, Durbán M (2015) Twenty years of p-splines. SORT Stat Oper Res Trans 39(2):0149–0186

[CR18] Endelman JB (2023) Fully efficient, two-stage analysis of multi-environment trials with directional dominance and multi-trait genomic selection. Theor Appl Genet 136(4):65 36949348 10.1007/s00122-023-04298-x PMC10033618

[CR19] Endelman JB, Atlin GN, Beyene Y, Semagn K, Zhang X, Sorrells ME, Jannink J-L (2014) Optimal design of preliminary yield trials with genome-wide markers. Crop Sci 54(1):48–59

[CR20] Federer WT (1956) Augmented (or hoonuiaku) designs

[CR21] Feoktistov V, Pietravalle S, Heslot N (2017). Optimal experimental design of field trials using differential evolution. In: 2017 IEEE congress on evolutionary computation (CEC). IEEE, pp 1690–1696

[CR22] Fernández-González J, Akdemir D, Isidro y Sánchez J (2023) A comparison of methods for training population optimization in genomic selection. Theor Appl Genet 136(3):30 36892603 10.1007/s00122-023-04265-6 PMC9998580

[CR23] Fernández-González J, Sánchez IY, J, (2025) Maximizing the accuracy of genetic variance estimation and using a novel generalized effective sample size to improve simulations. Theor Appl Genet 138(4):78 10.1007/s00122-025-04861-8 PMC11919955 40102234

[CR24] Fernández-González J, Isidro y Sánchez J (2025) Optimizing fully-efficient two-stage models for genomic selection using open-source software. Plant Methods 21(1):9 39905443 10.1186/s13007-024-01318-9 PMC11796230

[CR25] Ferreira Coelho I, Peixoto MA, Marcal TS, Bernardeli A, Silva Alves R, de Lima RO, Reis EF, Bhering LL (2021) Accounting for spatial trends in multi-environment diallel analysis in maize breeding. PLoS ONE 16(10):e0258473 34673808 10.1371/journal.pone.0258473 PMC8530354

[CR26] Fisher RA (1926) The arrangement of field experiments. J Minist Agricult 33:503–515

[CR27] Gianola D, de Los CG (2008) Inferring genetic values for quantitative traits non-parametrically. Genet Res 90(6):525–540 10.1017/S0016672308009890 19123970

[CR28] Gilmour AR, Cullis BR, Verbyla AP (1997) Accounting for natural and extraneous variation in the analysis of field experiments. J Agricult Biol Environ Stat 269–293 10.2307/1400446

[CR29] Gomes DG (2022) Should i use fixed effects or random effects when i have fewer than five levels of a grouping factor in a mixed-effects model? PeerJ 10:e12794 35116198 10.7717/peerj.12794 PMC8784019

[CR30] Granato I, Fritsche-Neto R (2018) snpReady: preparing genotypic datasets in order to run genomic analysis.(R package version 0.9.6))

[CR31] Guo T, Yu X, Li X, Zhang H, Zhu C, Flint-Garcia S, McMullen MD, Holland JB, Szalma SJ, Wisser RJ, Yu J (2019) Optimal designs for genomic selection in hybrid crops. Mol Plant 12(3):390–401 30625380 10.1016/j.molp.2018.12.022

[CR32] Hallauer AR, Carena MJ, Miranda Filho J (2010) Quantitative genetics in maize breeding, vol 6. Springer, Berlin

[CR33] Henderson C (1975) Best linear unbiased estimation and prediction under a selection model. Biometrics 31(2):423–447 1174616

[CR34] Henderson C (1977) Best linear unbiased prediction of breeding values not in the model for records. J Dairy Sci 60(5):783–787

[CR35] Henderson C (1985) Best linear unbiased prediction of nonadditive genetic merits in noninbred populations. J Anim Sci 60(1):111–117

[CR36] Henderson CR et al (1984) Applications of linear models in animal breeding, vol 462. University of Guelph, Guelph

[CR37] Hill WG, Goddard ME, Visscher PM (2008) Data and theory point to mainly additive genetic variance for complex traits. PLoS Genet 4(2):e1000008 18454194 10.1371/journal.pgen.1000008 PMC2265475

[CR38] Hirsch CN, Foerster JM, Johnson JM, Sekhon RS, Muttoni G, Vaillancourt B, Peñagaricano F, Lindquist E, Pedraza MA, Barry K, de Leon N, Kaeppler SM, Buell CR (2014) Insights into the Maize Pan-Genome and Pan-Transcriptome. Plant Cell 26(1):121–135 24488960 10.1105/tpc.113.119982 PMC3963563

[CR39] Holland JB, Nyquist WE, Cervantes-Martínez CT, Janick J (2003) Estimating and interpreting heritability for plant breeding: an update. Plant Breed Rev 22:9–112

[CR40] Hu J, Seiler G, Kole C (2010) Genetics, genomics and breeding of sunflower. CRC Press, Boca Raton

[CR41] Jaiswal M, Shweta RK, Mishra A, Mourya A, Yaduvanshi N, Singh S (2022) Estimation of genetic variability, heritability and genetic advance for seed yield and its components in oat. (avena sativa l.) under highly alkaline conditions. J Agric Res Technol 47(2):226–232 10.22271/tpi.2022.v11.i9f.15266

[CR42] Khan A (2001) Yield performance, heritability and interrelationship in some quantitative traits in sunflower. Helia 34:35–40 https://www.cabidigitallibrary.org/doi/full/10.5555/20023001957

[CR43] Laloë D (1993) Precision and information in linear models of genetic evaluation. Genet Sel Evol 25(6):557–576

[CR44] Lee D-J, Durbán M, Eilers P (2013) Efficient two-dimensional smoothing with p-spline anova mixed models and nested bases. Comput Stat Data Anal 61:22–37

[CR45] Lorenz AJ, Chao S, Asoro FG, Heffner EL, Hayashi T, Iwata H, Smith KP, Sorrells ME, Jannink J-L (2011) Genomic selection in plant breeding: knowledge and prospects. Adv Agron 110:77–123

[CR46] Lush J (1943) Animal breeding plans. Number Edn 2, Chapter 12. Iowa State College Press,(See chapter 12)

[CR47] Martin R, Eccleston J (1992) Recursive formulae for constructing block designs with dependent errors. Biometrika 79(2):426–430

[CR48] Melchinger AE, Frisch M (2023) Genomic prediction in hybrid breeding: Ii. Reciprocal recurrent genomic selection with full-sib and half-sib families. Theor Appl Genet 136(9):203 10.1007/s00122-023-04446-3 PMC10471712 37653062

[CR49] Meuwissen THE, Hayes BJ, Goddard ME (2001) Prediction of total genetic value using genome-wide dense marker maps. Genetics 157(4):1819–1829 11290733 10.1093/genetics/157.4.1819 PMC1461589

[CR50] Muñoz PR, Resende MF Jr, Gezan SA, Resende MDV, de Los CG, Kirst M, Huber D, Peter GF (2014) Unraveling additive from nonadditive effects using genomic relationship matrices. Genetics 198(4):1759–1768 25324160 10.1534/genetics.114.171322 PMC4256785

[CR51] Pfahler P (1971) Heritability estimates for grain yield in oats (Avena sp.). Crop Sci 11(3):378–381

[CR52] Piepho H-P, Möhring J, Schulz-Streeck T, Ogutu JO (2012) A stage-wise approach for the analysis of multi-environment trials. Biom J 54(6):844–860 10.1002/bimj.201100219 23007738

[CR53] Rincent R, Charcosset A, Moreau L (2017) Predicting genomic selection efficiency to optimize calibration set and to assess prediction accuracy in highly structured populations. Theor Appl Genet 130(11):2231–2247 28795202 10.1007/s00122-017-2956-7 PMC5641287

[CR54] Rincent R, Laloë D, Nicolas S, Altmann T, Brunel D, Revilla P, Rodríguez V, Moreno-Gonzalez J, Melchinger A, Bauer E, Schoen C-C, Meyer N, Giauffret C, Bauland C, Jamin P, Laborde J, Monod H, Flament P, Charcosset A, Moreau L (2012) Maximizing the reliability of genomic selection by optimizing the calibration set of reference individuals: comparison of methods in two diverse groups of maize inbreds (zea mays l. Genetics 192(2):715–728 22865733 10.1534/genetics.112.141473 PMC3454892

[CR55] Rodríguez-Álvarez MX, Boer MP, van Eeuwijk FA, Eilers PH (2017) Correcting for spatial heterogeneity in plant breeding experiments with p-splines. Spatial Stat 23:52–71

[CR56] Sánchez JI, Jannink JL, Akdemir D, Poland J, Heslot N, Sorrells ME (2015) Training set optimization under population structure in genomic selection. Theor Appl Genet 128(1):145–158 25367380 10.1007/s00122-014-2418-4 PMC4282691

[CR57] Stefanova KT, Smith AB, Cullis BR (2009) Enhanced diagnostics for the spatial analysis of field trials. J Agric Biol Environ Stat 14:392–410

[CR58] Tanvi K, Jindal Y (2017) Estimates of genetic variability, heritability and genetic advance in oats (Avena sp.) for seed and fodder yield traits. Forage Res 43(2):110–115

[CR59] Technow F, Riedelsheimer C, Schrag TA, Melchinger AE (2012) Genomic prediction of hybrid performance in maize with models incorporating dominance and population specific marker effects. Theor Appl Genet 125:1181–1194 22733443 10.1007/s00122-012-1905-8

[CR60] VanRaden P (2008) Efficient methods to compute genomic predictions. J Dairy Sci 91(11):4414–4423 18946147 10.3168/jds.2007-0980

[CR61] Vitezica ZG, Varona L, Legarra A (2013) On the additive and dominant variance and covariance of individuals within the genomic selection scope. Genetics 195(4):1223–1230 24121775 10.1534/genetics.113.155176 PMC3832268

[CR62] Welham SJ, Gogel BJ, Smith AB, Thompson R, Cullis BR (2010) A comparison of analysis methods for late-stage variety evaluation trials. Aust N Z J Stat 52(2):125–149

[CR63] Yue H, Gauch HG, Wei J, Xie J, Chen S, Peng H, Bu J, Jiang X (2022) Genotype by environment interaction analysis for grain yield and yield components of summer maize hybrids across the huanghuaihai region in china. Agriculture 12(5):602

[CR64] Zystro J, Colley M, Dawson J (2018) Alternative experimental designs for plant breeding. Plant Breed Rev 42:87–117

