# Supplementary material for: Unveiling the interactions between design optimization, modeling strategies, and spatial analysis in genomic selection
Source: Theor Appl Genet. 2026 Aug 28;139(9):249. doi: 10.1007/s00122-026-05285-8 (PMC13524979; doi:10.1007/s00122-026-05285-8)
Supplement: Supplementary file 1 [file 122_2026_5285_MOESM1_ESM.pdf]

Supplementary material

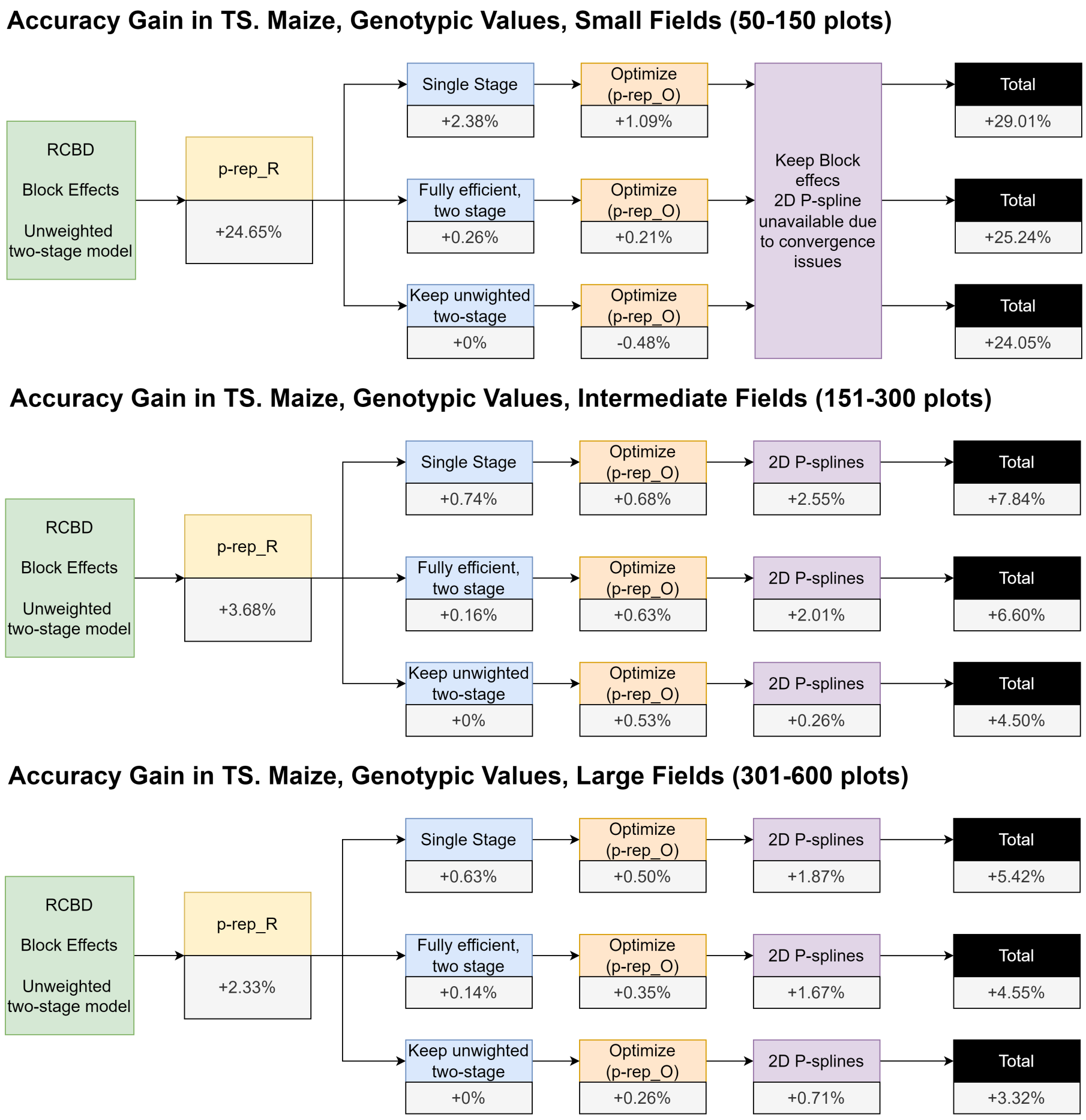

Fig. S1 Changes in prediction accuracy on the test set (TS) for genotypic values (additive + non-additive) in the Maize dataset as increasingly complex experimental designs and modeling are sequentially implemented.

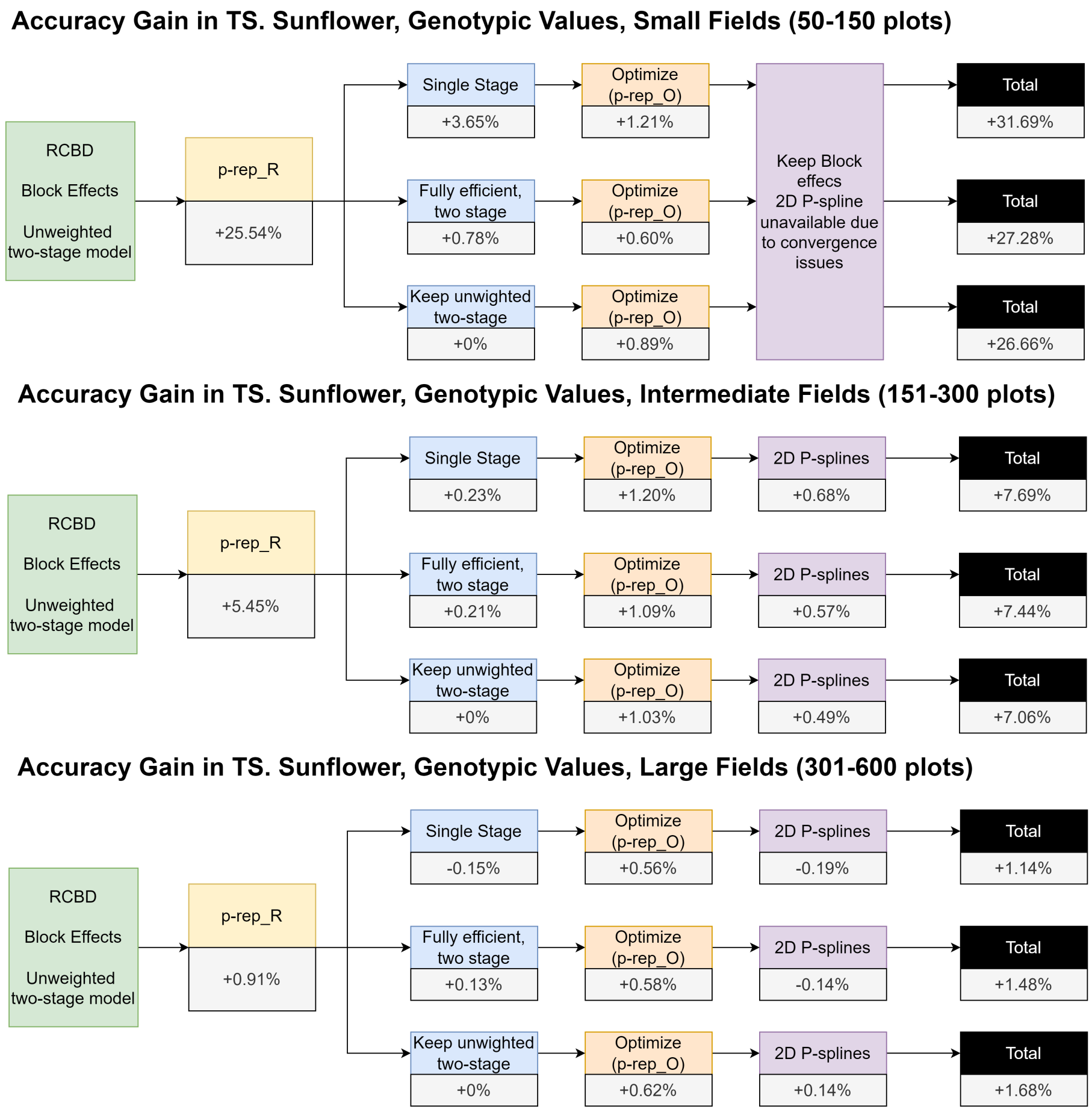

Fig. S2 Changes in prediction accuracy on the test set (TS) for genotypic values (additive + non-additive) in the Sunflower dataset as increasingly complex experimental designs and modeling are sequentially implemented.

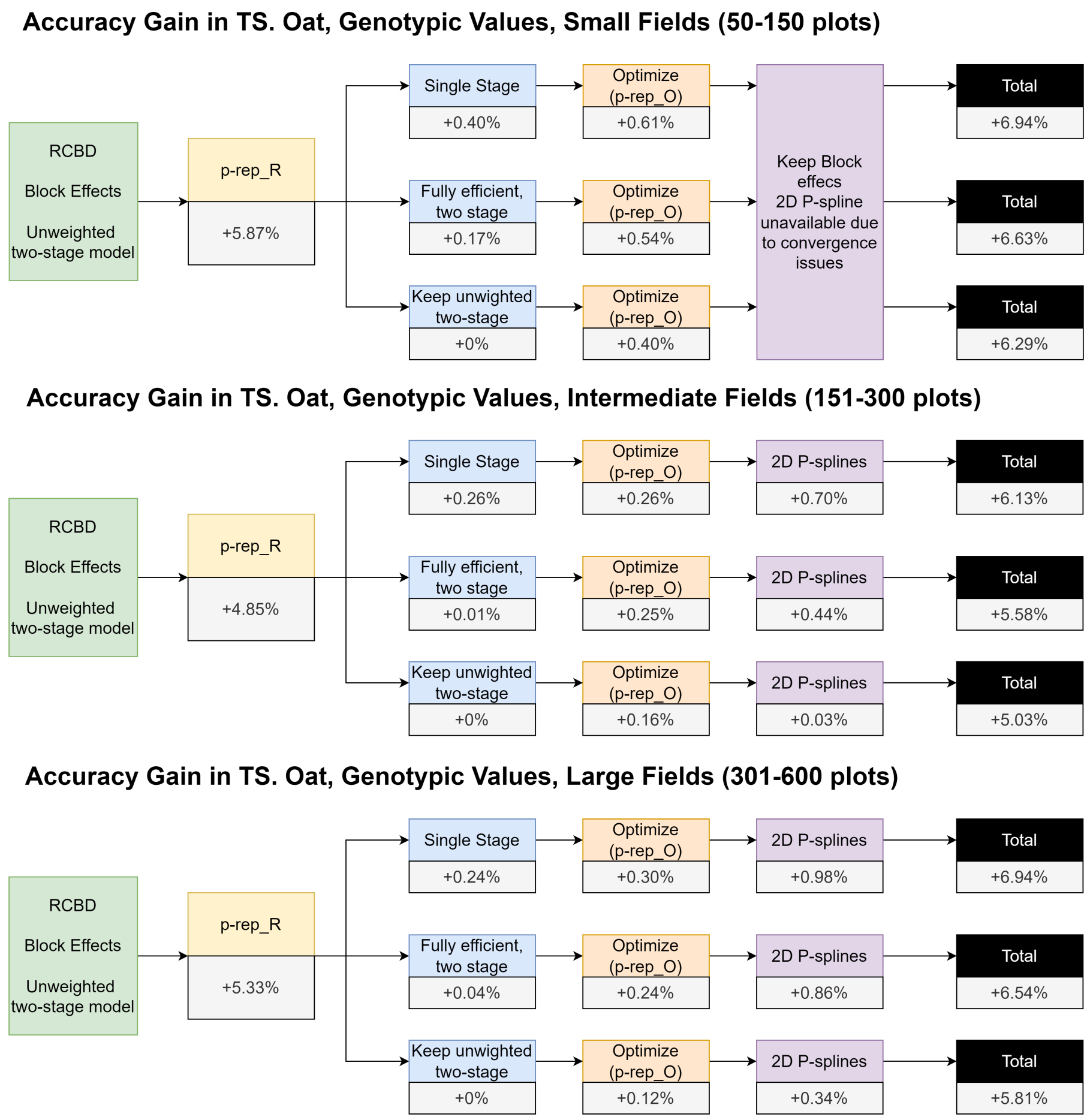

Fig. S3 Changes in prediction accuracy on the test set (TS) for genotypic values (additive + non-additive) in the Oat dataset as increasingly complex experimental designs and modeling are sequentially implemented.

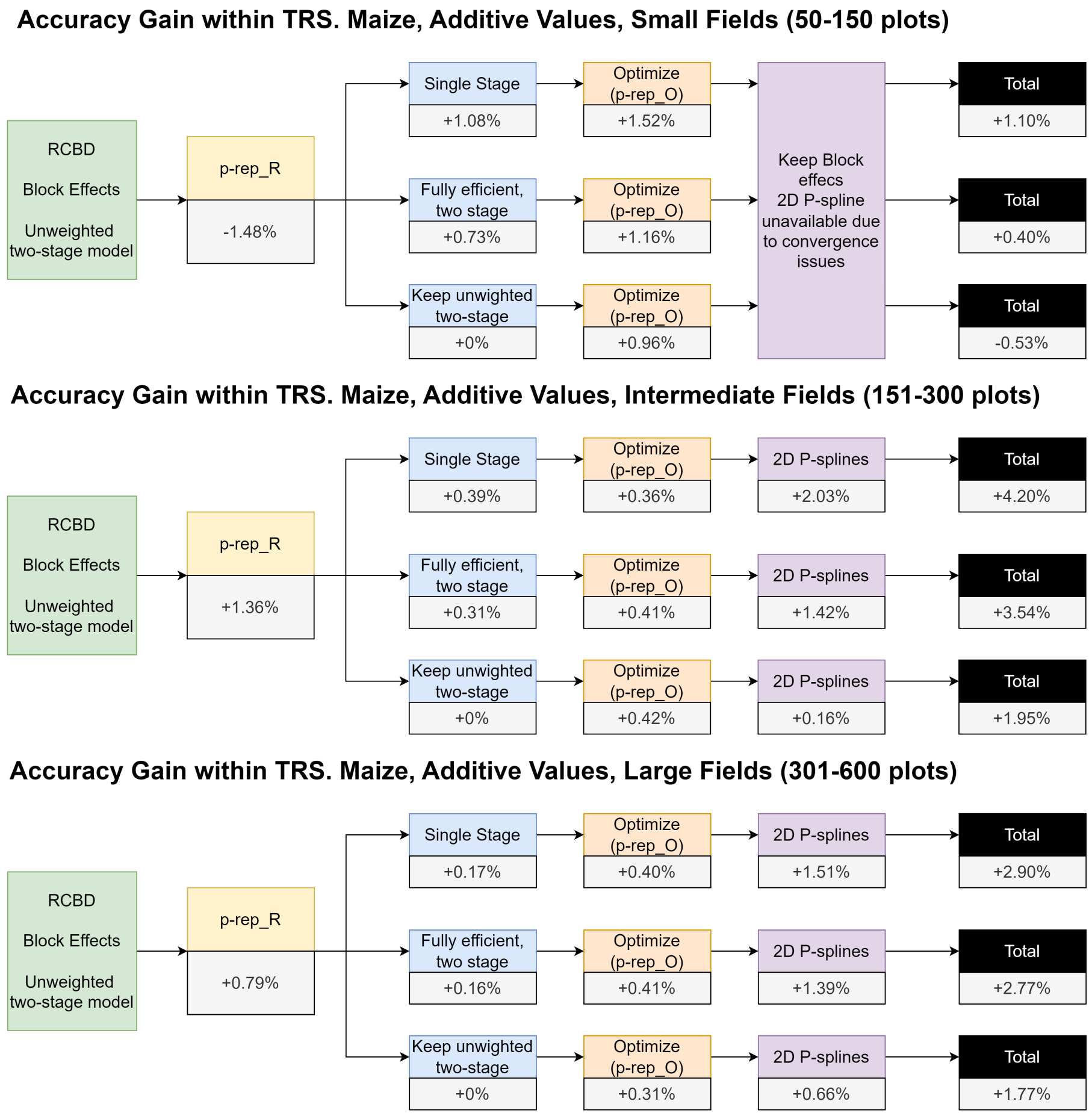

Fig. S4 Changes in within-training set (TRS) prediction accuracy for additive genetic values in the Maize dataset as increasingly complex experimental designs and modeling are sequentially implemented.

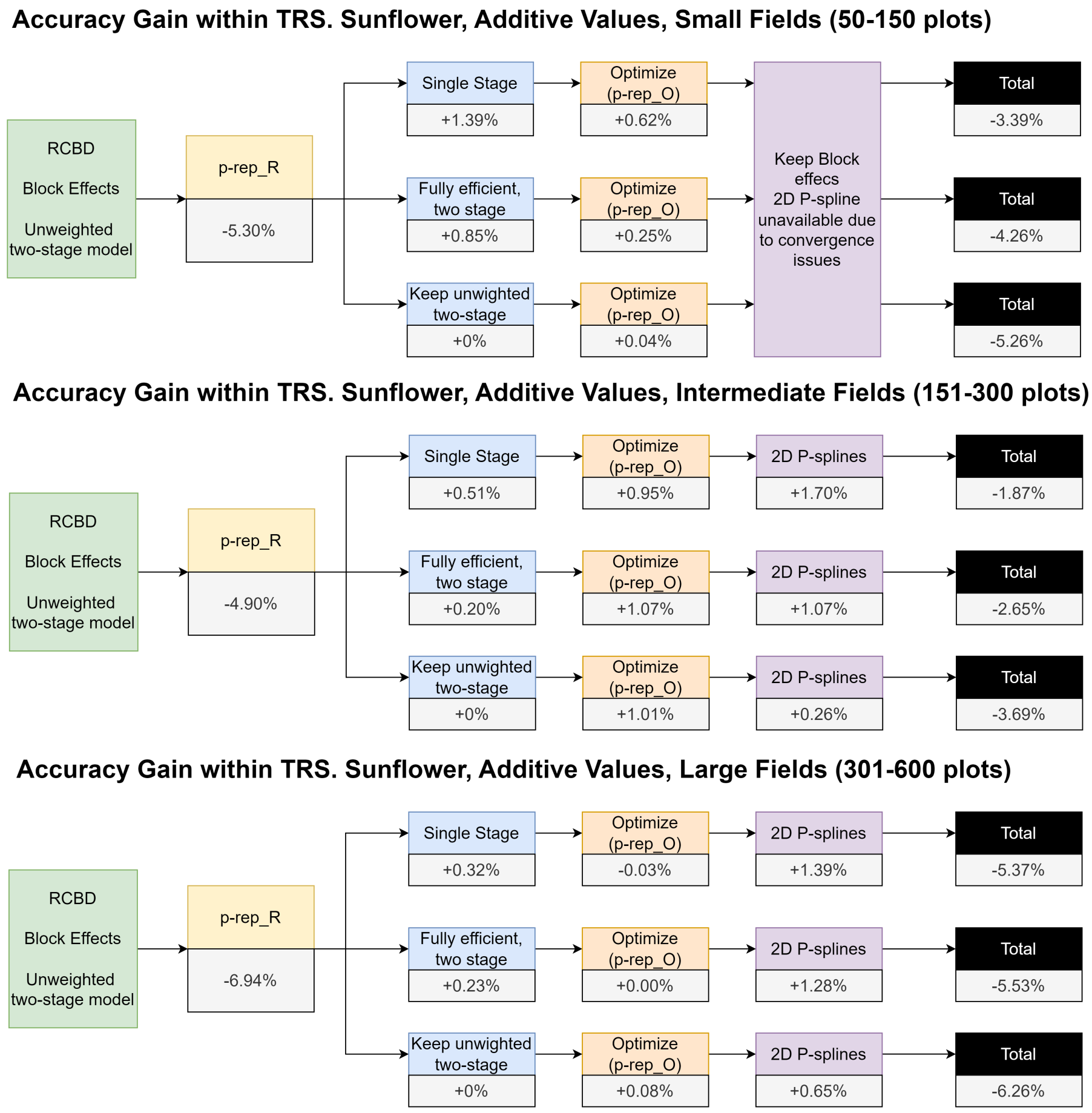

Fig. S5 Changes in within-training set (TRS) prediction accuracy for additive genetic values in the Sunflower dataset as increasingly complex experimental designs and modeling are sequentially implemented.

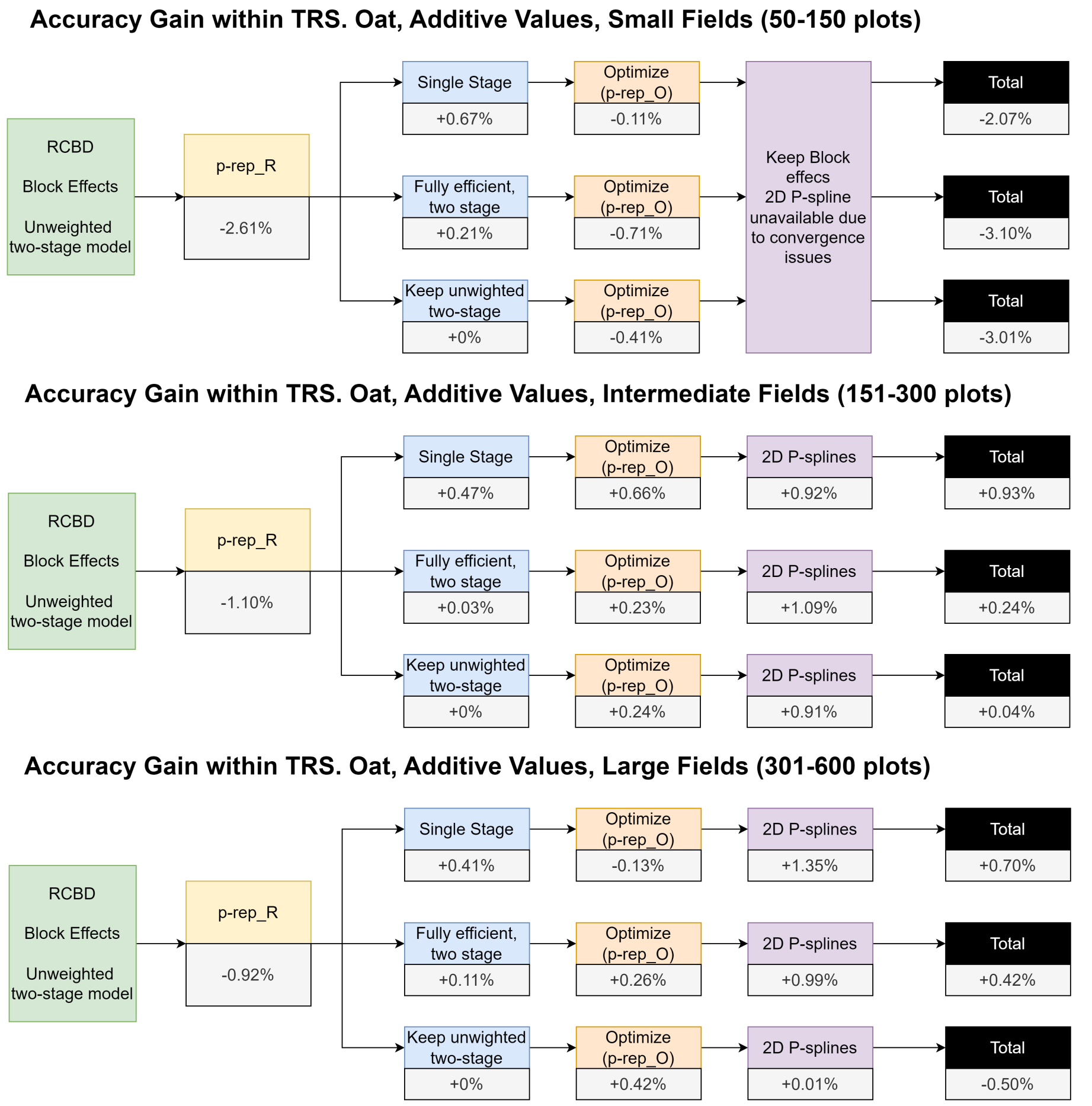

Fig. S6 Changes in within-training set (TRS) prediction accuracy for additive genetic values in the Oat dataset as increasingly complex experimental designs and modeling are sequentially implemented.

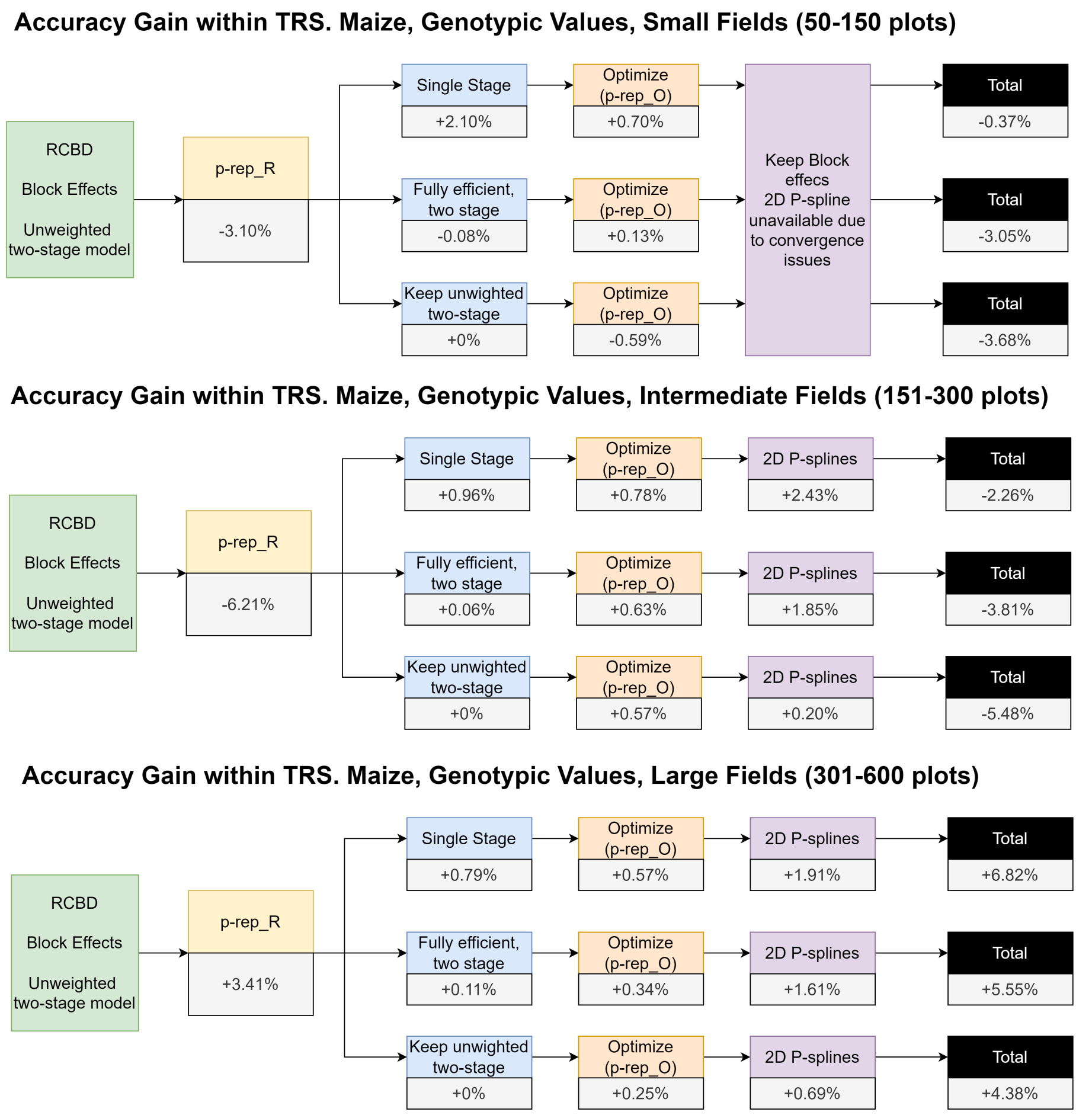

Fig. S7 Changes in within-training set (TRS) prediction accuracy for genotypic values (additive + non-additive) in the Maize dataset as increasingly complex experimental designs and modeling are sequentially implemented.

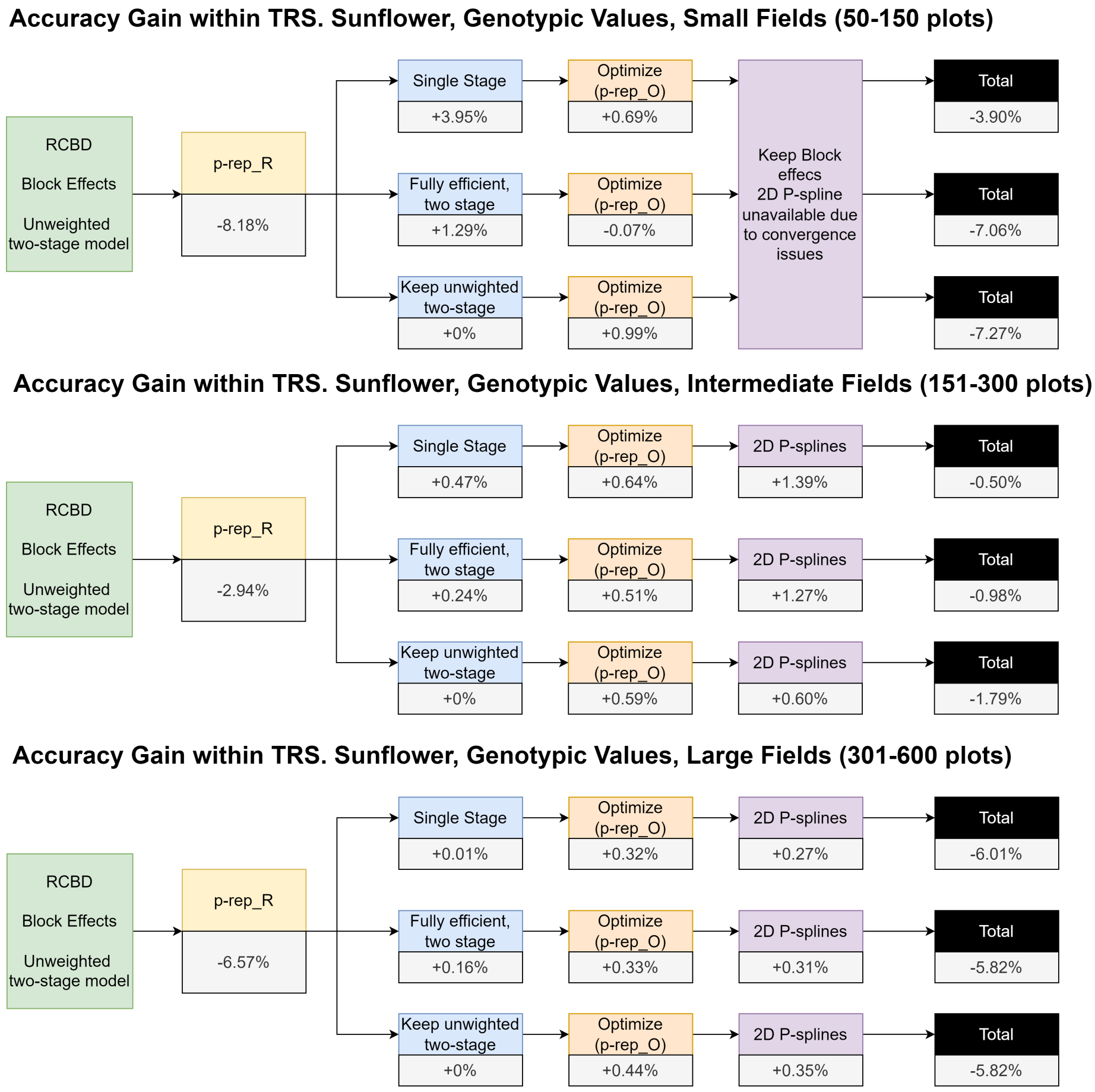

Fig. S8 Changes in within-training set (TRS) prediction accuracy for genotypic values (additive + non-additive) in the Sunflower dataset as increasingly complex experimental designs and modeling are sequentially implemented.

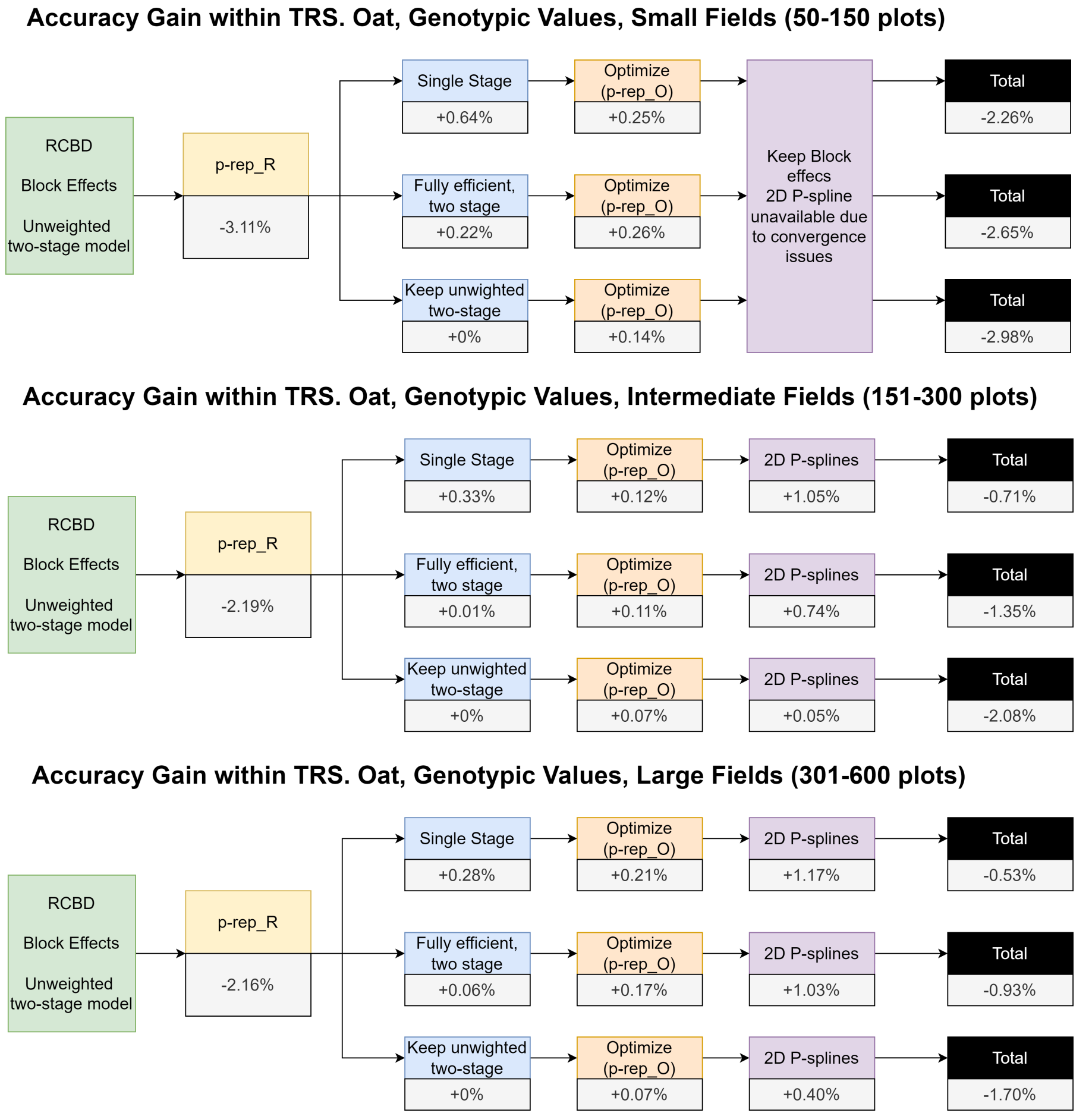

Fig. S9 Changes in within-training set (TRS) prediction accuracy for genotypic values (additive + non-additive) in the Oat dataset as increasingly complex experimental designs and modeling are sequentially implemented.
